# Intervention Now to Eliminate Repeat Unintended Pregnancy in Teenagers (INTERUPT): a systematic review of intervention effectiveness and cost-effectiveness, and qualitative and realist synthesis of implementation factors and user engagement

**DOI:** 10.1186/s12916-017-0904-7

**Published:** 2017-08-15

**Authors:** Rabeea’h W. Aslam, Maggie Hendry, Andrew Booth, Ben Carter, Joanna M. Charles, Noel Craine, Rhiannon Tudor Edwards, Jane Noyes, Lupetu Ives Ntambwe, Diana Pasterfield, Jo Rycroft-Malone, Nefyn Williams, Rhiannon Whitaker

**Affiliations:** 1 Department of Biostaistics, Institute of Translational Medicine, University of Liverpool, Liverpool, UK; 2 North Wales Centre for Primary Care Research School of Healthcare Sciences, Bangor University, Bangor, UK; 3 School of Health and Related Research (ScHARR), University of Sheffield, Sheffield, UK; 4 Department of Biostatistics and Health Informatics, Institute of Psychiatry Psychology & Neuroscience, King’s College London, London, UK; 5 Centre for Health Economics and Medicines Evaluations, School of Healthcare Sciences, Bangor University, Bangor, UK; 6 Public Health Wales, Bangor, UK; 7 School of Social Sciences, Bangor University, Bangor, UK; 8 Veristat, Montreal, Canada; 9 Centre for Health-Related Research School of Healthcare Sciences, Bangor University, Bangor, UK; 10 Whitaker Research Ltd., Bangor, UK

**Keywords:** Pregnancy, Adolescent, Complex interventions, Contraceptives, Prevention, Childbearing

## Abstract

**Background:**

Unintended repeat conceptions can result in emotional, psychological and educational harm to young women, often with enduring implications for their life chances. This study aimed to identify which young women are at the greatest risk of repeat unintended pregnancies; which interventions are effective and cost-effective; and what are the barriers to and facilitators for the uptake of these interventions.

**Methods:**

We conducted a mixed-methods systematic review which included meta-analysis, framework synthesis and application of realist principles, with stakeholder input and service user feedback to address this. We searched 20 electronic databases, including MEDLINE, Excerpta Medica database, Applied Social Sciences Index and Abstracts and Research Papers in Economics, to cover a broad range of health, social science, health economics and grey literature sources. Searches were conducted between May 2013 and June 2014 and updated in August 2015.

**Results:**

Twelve randomised controlled trials (RCTs), two quasi-RCTs, 10 qualitative studies and 53 other quantitative studies were identified. The RCTs evaluated psychosocial interventions and an emergency contraception programme. The primary outcome was repeat conception rate: the event rate was 132 of 308 (43%) in the intervention group versus 140 of 289 (48%) for the control group, with a non-significant risk ratio (RR) of 0.92 [95% confidence interval (CI) 0.78–1.08]. Four studies reported subsequent birth rates: 29 of 237 (12%) events for the intervention arm versus 46 out of 224 (21%) for the control arm, with an RR of 0.60 (95% CI 0.39–0.93). Many repeat conceptions occurred in the context of poverty, low expectations and aspirations and negligible opportunities. Qualitative and realist evidence highlighted the importance of context, motivation, future planning and giving young women a central and active role in the development of new interventions.

**Conclusions:**

Little or no evidence for the effectiveness or cost-effectiveness of any of the interventions to reduce repeat pregnancy in young women was found. Qualitative and realist evidence helped to explain gaps in intervention design that should be addressed. More theory-based, rigorously evaluated programmes need to be developed to reduce unintended repeat pregnancy in young women.

**Trial registration:**

PROSPERO, CRD42012003168. Cochrane registration number: i = fertility/0068

**Electronic supplementary material:**

The online version of this article (doi:10.1186/s12916-017-0904-7) contains supplementary material, which is available to authorized users.

## Background

Repeat pregnancy in adolescents is a public health concern across the world, since it frequently occurs in the context of economic constraints and poor maternal and child well-being [1–3]. Although the rates are continuing to fall, the UK has the fourth highest rate in western Europe, and one-fifth of these pregnancies are estimated to be repeat pregnancies [3–8]. Around three-quarters of adolescent pregnancies are unplanned with up to a half resulting in abortion [9–11]. Unintended conceptions can present enduring emotional, psychological and educational challenges, as well as implications for the life opportunities of young mothers and their children [9, 10, 12]. It is important to identify effective and cost-effective strategies that are acceptable to young women [13–15].

Repeat pregnancy is defined here as the incidence of two or more pregnancies before the age of 20 years. ‘Unintendedness’ is defined as any incidence of pregnancy when intention was not specifically stated [16]. The social predictors of repeat adolescent pregnancy are varied at individual, couple, family, community and social levels [17] and are similar to those of first pregnancies in young women [18].

Local and national public health programmes in different countries have tried to address the short- and long-term consequences of unintended adolescent pregnancies [9, 11–14, 19, 20]. Some complex interventions which have focussed on sex education, skills training for jobs and personal development for young women are effective at reducing first pregnancies [21–23]. However, it is not clear whether these interventions are effective at preventing repeat unintended pregnancies.

## Objectives

Our objectives were to identify which young women were at the greatest risk of repeat unintended pregnancies; which interventions were effective and cost-effective; and barriers to, and facilitators of, the uptake of these interventions.

## Methods

**Fig. 1:**
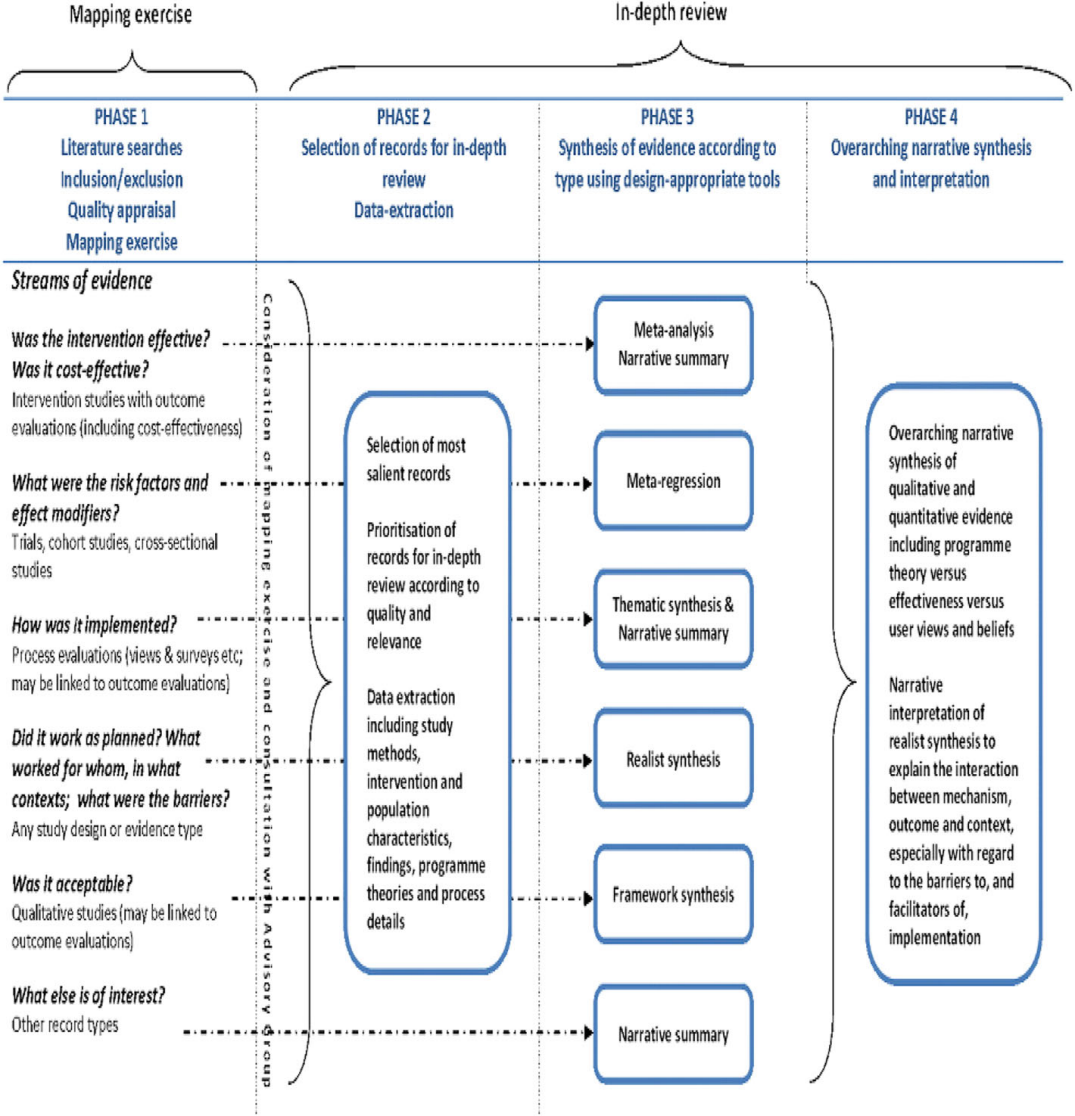
Overview of review methods

**Fig. 2:**
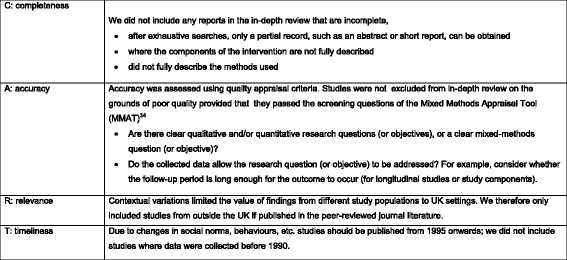
CART criteria

We conducted a multi-streamed, mixed-methods systematic review, guided by an advisory group of stakeholders, which followed the Preferred Reporting Items for Systematic Reviews and Meta-Analyses (PRISMA) checklist. We used a structured, iterative approach combining methods tailored to each stream of evidence (Fig. 1). A mapping exercise and the study selection were based on the approach used by the Evidence for Policy and Practice Information and Co-ordinating Centre (EPPI-Centre) [24]. First, extensive literature searches were conducted, and the evidence was screened against explicit inclusion and exclusion criteria. Then two initial screening criteria from the Mixed-Methods Appraisal Tool (MMAT) [25] were applied to establish that all included studies had clear research questions or objectives that could be addressed by the data collected. In Phase 1 of the review, a mapping exercise was undertaken to organise and describe the evidence for a clear picture of the body of research. These findings of the mapping exercise and possible gaps in the evidence were then presented to the service provider consultation group. In Phase 2, studies were selected for in-depth review and data extraction based on a Completeness, Accuracy, Relevance and Timeliness (CART) framework (Fig. 2) to choose the best evidence for in-depth review [26, 27]. During Phase 3 of this review, the evidence was synthesised based on study type and using design-specific methods. The quantitative data were synthesised with reference to Cochrane guidelines for effectiveness studies [28], qualitative evidence was synthesised using Framework methods [29] and principles of realist synthesis were used to uncover theories and mechanisms underpinning interventions [30]. Members of the review team presented preliminary findings at a second meeting of the service provider consultation group and subsequently to a group of young women who had experience of adolescent pregnancy and early parenthood at meetings facilitated by staff members of a support group for young mothers called Flying Start. Finally, an overarching narrative summary of the evidence was produced during Phase 4. Figure 1 shows a schematic overview of our review methods, which are described in detail in a published protocol [27].

In Phase 1 of the study, we identified the literature, a brief quality appraisal and mapped the evidence

### Data sources

We searched 20 electronic databases, including MEDLINE, Excerpta Medica database (Embase), the Cochrane Library, Applied Social Sciences Index and Abstracts (ASSIA) and Research Papers in Economics (RePEc), to cover a range of health, social science, health economics and grey literature sources (full details are reported in the protocol [27]). Searches were conducted between May 2013 and June 2014 and updated in August 2015. The searches were limited to articles published from 1990 onwards as the stakeholder group advised us that literature published earlier than this would not be relevant, but no language restrictions were applied. Examples of the MEDLINE search strategy and hand-searched journals are given in Additional file 1: Section 1.

### Outcomes of interest

The primary outcomes for reducing repeat unintended pregnancies in young women were:

- Effectiveness of interventions (unintended pregnancy in young women, measured as the difference in proportion of girls who go on to have a repeat pregnancy)
- Acceptability of interventions (the proportion of participants who reported the intervention was acceptable, or in absence of this, the proportion of participants who were willing to be recruited into the study)

The phenomena of interest for the qualitative synthesis and realist review were:

- Views and experiences of young mothers, families and professionals
- Identification of barriers and facilitators of interventions relating to acceptability, uptake and feasibility of implementation
- Programme theories that suggest the mechanism by which the intervention is expected to work

In Phase 2 of the study, we selected and prioritised the evidence for in-depth review and data extraction.

### Study selection

Out of the 8668 studies identified, the inclusion criteria (i.e. studies of any design, from any country or in any language, which focussed on interventions for, views on or risk factors for repeat adolescent pregnancy) were applied to 5783 titles and abstracts after duplicates were removed. We assessed 232 full-text articles for eligibility, and 118 studies were included in the initial mapping exercise.

The study characteristics, including study methods, context, participants and interventions, were identified. We presented this information to our stakeholder group at a workshop and invited their views on how the review should be focussed. Based on their feedback, and in view of the large number of studies, we applied the CART framework (Additional file 1: Section 2) to choose the best evidence for in-depth review [26, 27].

### Assessing the quality of evidence

The Cochrane Risk of Bias tool was used to assess the quality for randomised controlled trials (RCTs). The screening questions from the MMAT were applied to all the studies during the mapping phase, and the questions appropriate to qualitative data were used to appraise the qualitative studies [25]. The Cochrane Risk of Bias tool was used for randomised trials [28] and the Drummond [31] checklist for economic evidence. The Grading of Recommendations Assessment, Development and Evaluation (GRADE) approach was used to evaluate the certainty of the findings from the RCTs [32], the Confidence in the Evidence from Reviews of Qualitative research (CERQual) approach was used for qualitative studies [33] and criteria adapted from Pawson [30] were used for the realist synthesis.

### Data extraction

We have presented the findings of the data extraction exercise in a table of study characteristics, which include the study details, setting, population, quality score, methods, etc. We also presented sociodemographic characteristics known to be important from an equity perspective. For this process, the PROGRESS (Place, Race, Occupation, Gender, Religion, Education, Socioeconomic status (SES), Social capital) framework was utilised [34].

Data extraction forms were developed and piloted in Microsoft Access using a sample of included studies. Data were extracted by one reviewer and independently checked for accuracy by a second reviewer, with disagreements resolved through discussion with a third reviewer where necessary.

During Phase 3, we synthesised the evidence based on type of evidence. Figure 1 illustrates the method of synthesis utilised for each evidence type.

#### Meta-analysis

The quantitative data were synthesised with reference to Cochrane guidelines for effectiveness studies [28]. Where possible, data have been pooled using a random effects model with an inverse variance method. Heterogeneity has been summarised using the *I* ^*2*^ summary metric [28]. Subgroups were explored to explain severe heterogeneity.

#### Sensitivity analyses

Firstly, only studies with a low and unclear risk of bias were included for a sensitivity analysis to investigating sources of heterogeneity. Secondly to evaluate all of the data for the primary outcome, a sensitivity analysis was carried out including quasi-experimental and observations studies.

#### Qualitative synthesis

For qualitative studies, or qualitative elements in mixed-method studies, we used the Framework method described by Ritchie and Spencer [29]. We used an a priori coding framework adapted from the Support Unit for Research Evidence (SURE) checklist for identifying factors affecting the implementation of a policy option [33, 35].

#### Realist synthesis

We selected subsets of evidence and applied the principles of realist synthesis [30]. From this stream of work a paper has already been published [36], which identified explicit or implicit theories by postulating how an intervention has an underlying causal mechanism that works in a defined social context to result in a particular outcome. These theories were used to explain the failure of an intervention to work. Additional theories were also identified from the wider literature (e.g. policy documents), the advisory group members or personal contact with other experts in the field. Data synthesis involved individual reflection and team discussion to examine the integrity of each theory [30]. Coded data from the studies were then used to confirm, refute or refine the candidate theories.

#### Cost-effectiveness

We provided a narrative review of economic evaluations of interventions specifically designed to address the issue in question.

During Phase 4, we combined all the evidence streams and interpreted the findings in an overarching narrative synthesis; we juxtaposed the programme theories of interventions from the trials evidence against the qualitative synthesis, risk factors, realist synthesis and views of stakeholders and service users [24, 27–30].

#### Stakeholder engagement and Patient and Public Involvement

Members of the research team were involved in co-ordinating a project, Empower to Choose, targeting repeat conceptions in young mothers, which is part of the Welsh Government’s Sexual Health and Wellbeing Action Plan12 aimed at reducing the rate of unwanted pregnancies in young mothers. Empower to Choose was guided by the Task and Finish Group, which included a group of practitioners, stakeholders (including public health, primary care, sexual health, obstetrics and midwifery representatives), policy makers and academics. This group was coordinated by Public Health Wales (PHW). Members of the Task and Finish Group were engaged in Phases 1 and 3. In Phase 3, different interventions and analysis from the review were also presented in a separate meeting to a group of 17 young mothers ranging in age from 15 to 22 years, with the assistance of two frontline organisations, Barnardo’s Cymru (Cardiff, UK) and Flying Start (Swansea, UK) [25–28, 30–33].

## Results

### Identified studies and risk of bias

**Fig. 3:**
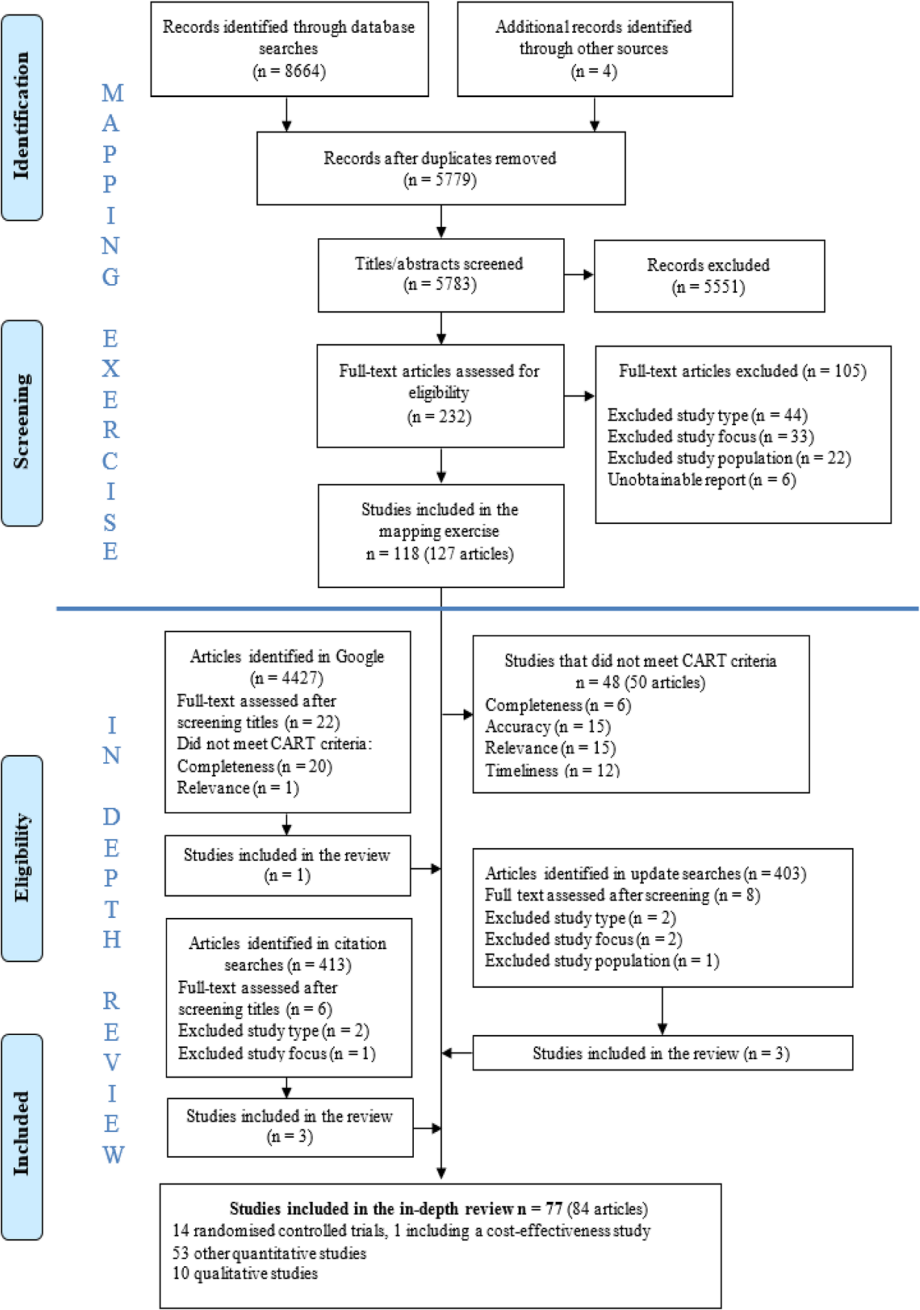
PRISMA flow diagram

Search results and study selection are summarised in a PRISMA flow diagram (see Fig. 3).

Seventy-seven studies were eligible to be included in the in-depth review. These comprised 12 RCTs [9, 19, 37–46] (one with an economic evaluation) [10], two quasi-RCTs [19, 47] and 10 qualitative studies [20, 48–57]. The study characteristics have been summarised in Additional file 1: Section 3 and Section 4. There were also 53 other quantitative studies, but since they tended to be of poor quality and their findings were relatively inconclusive, they made no useful contribution to the review. We analysed 10 individually randomised trials in the primary meta-analysis [9, 10, 19, 20, 37, 39–46]. Of the remaining trials, one was a cluster randomised trial [47], one was an analysis of smaller unpublished randomised and quasi-randomised trials and had an uncertain risk of bias [58] and two studies [59, 60] were non-randomised trials. These four studies were included in the sensitivity analysis. The quality assessments of the included quantitative and qualitative studies using these tools are presented in Additional file 1: Section 5, Section 6 and Section 7.

### Quality of evidence

Of the trials, five studies had a high risk of bias [9, 11, 38–40, 46], three studies had a low risk of bias [19, 20, 37, 43] and four studies [41, 42, 44, 45] had an unknown risk of bias. We additionally applied the GRADE approach [32] to judge the quality of the overall evidence for each outcome (Additional file 1: Section 6), and rated the outcomes for each intervention as moderate quality.

Using the MMAT tool [25] and considering the extent to which findings were supported by extracts from the original data (i.e. “thickness” and “richness”), we judged the qualitative evidence to be of moderate to high quality. Applying the CERQual approach (Additional file 1: Section 7), based on the methodological limitations of the individual studies and the coherence of each finding [61], our confidence in the certainty of findings from the qualitative synthesis was high (18 findings) to moderate (4 findings) with three findings of low certainty because they were only found in one study and either lacked supporting data or the finding itself was equivocal.

The results are described in the following sections, using the two main types of interventions from the trials, and juxtaposing the contextual detail for these interventions from the qualitative studies, with an explanation of why these interventions work (or don’t work), using realist principles.

### Interventions

The trial interventions fell into two broad categories: multi-element psychosocial interventions and a contraceptive programme.

#### Psychosocial interventions

The psychosocial programmes offered diverse services, such as case management and referral; education about pregnancy, labour and delivery, contraception and infant health; child developmental training; contact facilitation with the health-care system; and individual counselling. Most of these programmes involved home visits [19, 20], two were community based [40, 46] and one involved telephone counselling [42]. Follow-up periods also ranged from 12 months [42] to 24 months [19].

##### Home-based interventions

**Fig. 4:**
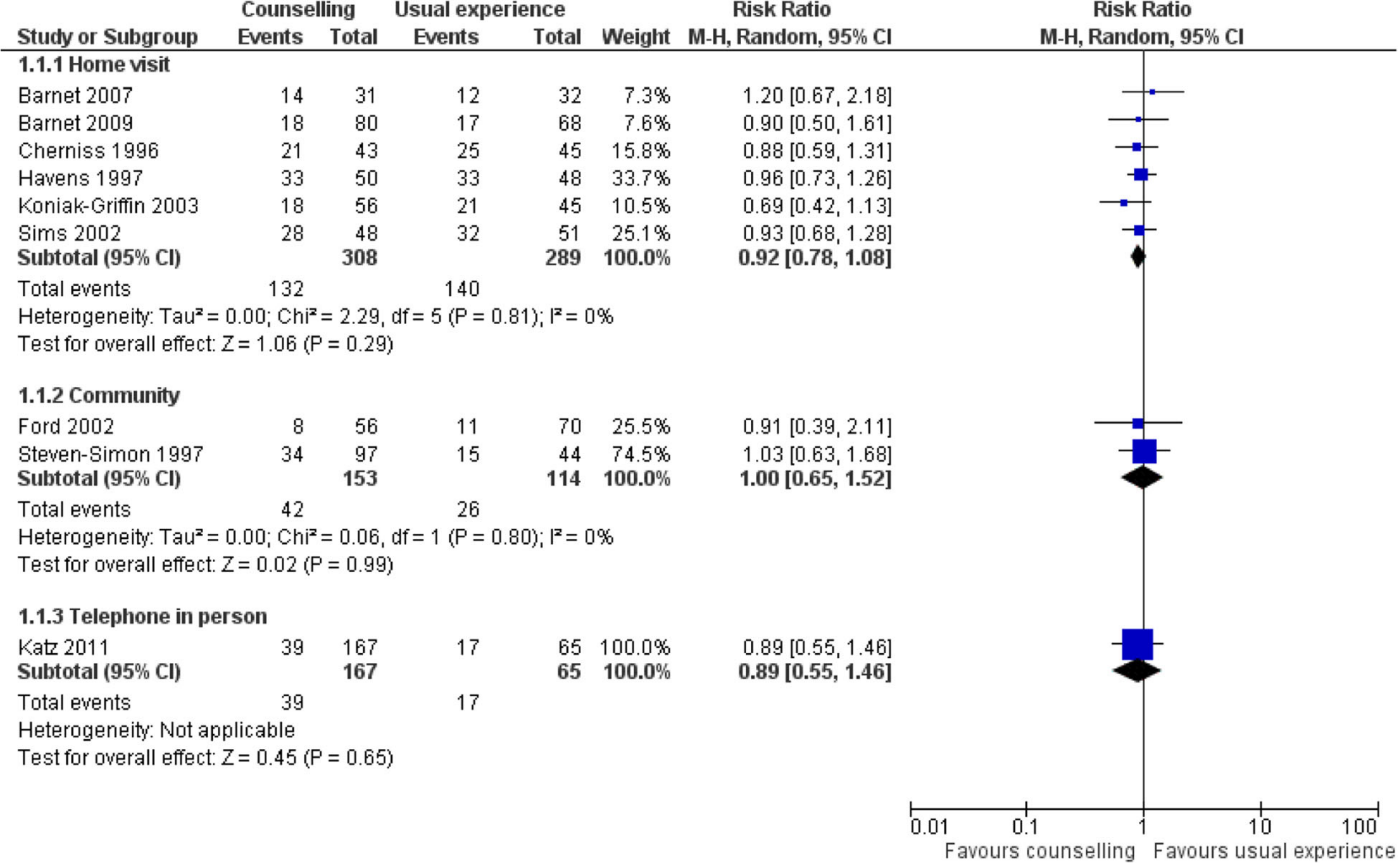
Forest plot of comparison (proportion of repeat pregnancy)

The interventions based on home visits had counsellors [19, 20], mentors [38, 46], midwives [44], nurses [43] or trained home visitors [19, 20] delivering the interventions to young mothers at their homes. These professionals [43] and paraprofessionals [45] could be state-sponsored, recruited from the community [19, 20] or from the same ethnic group [42]. All six trials [19, 20, 38, 39, 43, 45, 46] of home-based psychosocial interventions reported on the effectiveness of the intervention in reducing the proportion of repeat pregnancies. The combined event rate was 132 of 308 for the intervention arm versus 140 of 289 for the control arm, giving a non-significant risk ratio (RR) of 0.92 [95% confidence interval (CI) 0.78–1.08]. None of the individual studies showed a significant effect (Fig. 4). However, when four larger, but lower quality studies [47, 58, 59] were included in the sensitivity analysis of the primary outcome (unintended repeat pregnancy), the estimate approached but did not reach statistical significance: event rates of 288 of 1077 (27%) in the intervention arm and 297 of 1004 (30%) in the control arm, giving an RR of 0.88 (95% CI 0.78–1.00). (See Additional file 1: Section 10.)

We advanced the programme theory that repeat home visits facilitate access to services, address gaps in social support networks and sustain behaviour change by repeated contact with young mothers, thereby directing them away from repeat pregnancy.

In a feedback session, young mothers stated a preference for home visits, since this approach allowed them to express their individual needs. Nonetheless, to increase the likelihood of this intervention working, realist theory suggests that the staff conducting home visits should have specialist training. Two of the biggest concerns of the health-care professionals in our consultation group were (1) the inconsistent knowledge base of the health-care professionals who provide advice on contraception for young adolescents and (2) the absence of life-skills training, making young mothers more susceptible to repeat pregnancies. The qualitative studies could shed no further light here since none was undertaken in the context of an intervention.

##### Community-based interventions

**Fig. 5:**
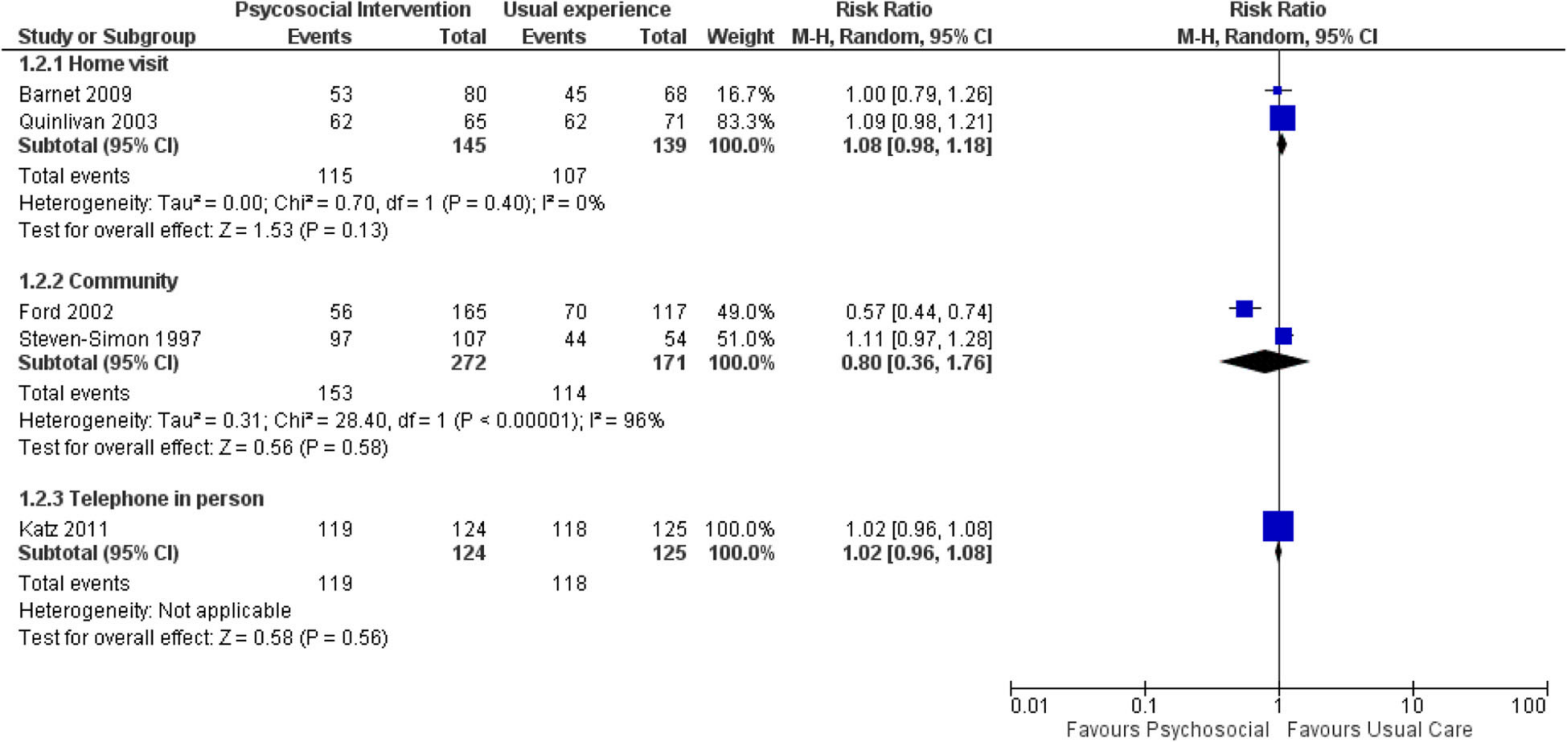
Forest plot od comparison (acceptablity of intervention)

Two trials of interventions based in the community, one of which involved a scheduled peer-centred prenatal care programme [40], and the other monetary incentives promoting mentor-led peer-support group participation [46], reported on their effectiveness in reducing repeat pregnancies. The combined event rate was 42 of 153 for the intervention arm versus 26 of 114 for the control arm, giving a non-significant risk ratio (RR) of 1.00 [0.65, 1.52) in favour of the intervention (Fig. 5).

These interventions did not reduce repeat conceptions in young women (GRADE rating of moderate), and there were no qualitative studies to support their approach. However, in feedback sessions, health professionals stated that transport to and from the location and the availability of food, refreshments and crèche facilities could all increase engagement and improve attendance rates. They also stated that using a ‘buddy system’ or peer support group could offer choices that empower young women and give them confidence, as well as giving them the opportunity to state what they want and need. The adolescent mothers in the service user group appreciated being part of a peer group.

The primary outcome, acceptability of the intervention (inferred as a proxy measure), from one study [40] showed significant differences between arms, but on combining the results with the other trial [46], there were no overall significant differences between arms.

##### Telephone-based interventions

One study reported a telephone-based mentoring intervention delivered by young female counsellors of similar ethnic backgrounds as the young women involved [42]. The event rate for the effectiveness was 39 of 167 for the intervention arm versus 17 out of 65 for the control arm. This gives a non-significant RR of 0.89 (0.55, 1.46) in favour of the intervention.

#### Contraceptive programme

The contraceptive programme offered education and advance provision of emergency contraception by a licensed health professional. The contraception intervention study showed a reduction in the number of repeat pregnancies in the intervention group (10 of 48) compared with the control group (14 of 43), giving an RR of 0.69 (95% CI 0.34–1.14); however, this was not statistically significant. Supplying emergency contraception is aimed at reducing repeat pregnancies by addressing frequent discontinuation or switching of contraceptive methods. Although not related to a specific intervention, the qualitative studies revealed some reasons why young women failed to maintain effective contraceptive use. Many women experienced side-effects with the more reliable methods. Women commonly stopped using one method before obtaining another, which rendered them vulnerable to unwanted pregnancy in the interim [49, 50, 56]. These women lacked basic knowledge about contraceptive methods [48, 55, 57]. There were common misconceptions, particularly about fertility soon after birth or when breastfeeding [49, 57] and about the side-effects of some types of contraception. Women also encountered significant barriers to accessing contraception, including restrictive clinic hours, patchy service provision and other system failures, such as lack of provider training [49, 50, 52, 56, 57].

Health-care professionals in our stakeholder group emphasised the disadvantages of using emergency contraceptives as the sole method of contraception. They also acknowledged that since long acting reversible contraceptives (LARCs) are not easily accessible through general practitioners, repeated appointments have to be made, which increases the susceptibility to repeat pregnancy. The service user group stated a preference for LARCs as they provided cover for a long period of time. They also highlighted the challenge presented by the 72-h requirement for emergency contraception, with bank holiday weekends or the prolonged Christmas break being a cause for concern. The user group told us women were also hesitant in asking for emergency contraception for fear of being judged.

The realist synthesis allowed the review team to identify contextual features of the included interventions and their underlying mechanisms such as connectedness and tailoring (Additional file 1: Section 9). The mechanism of connectedness through peer or mentor support may trigger self-determination and active control. Feeling connected and supported can help an adolescent feel that her life choices are being encouraged and that she is being heard. The mechanism of tailoring is evident through situating the intervention within a broad context, taking account of the adolescent’s life experiences, developmental stage, culture and experiences (including pregnancy). The review highlighted contraceptive methods and preferences, barriers and facilitators as ways to implement tailoring. An individual, holistic approach to care may be more successful than adopting a purely medical model of providing information and then encouraging the use of hormonal or long acting reversible methods. It is important to assess an adolescent’s knowledge of contraceptive methods and her individual preferences and needs. Furthermore, adolescents’ circumstances, including transport challenges and difficulties accessing services, needed consideration and a tailored approach. Facilitators such as home visits and school-based services could minimise travel and promote access. Incentives such as crèche facilities or transport could increase engagement with an intervention and attendance rates. The mechanisms uncovered could increase the likelihood of an intervention being effective in preventing rapid repeat pregnancy in adolescents, which has been further explored in a recently published paper [36].

### Cost-effectiveness

Only one economic evaluation, a cost-effectiveness analysis [10], was found. However, as the intervention associated with this cost-effectiveness analysis showed no effect, we cannot make definitive conclusions about the economic evidence relating to interventions designed to prevent repeat adolescent pregnancy.

### Risk factors for repeat pregnancy

Of the 53 quantitative studies which examined risk factors, most tended to be of poor quality, and their findings were relatively inconclusive. They examined and demonstrated no empirical evidence for the association between repeat unintended pregnancy and factors, such as age, education, history of abuse, smoking, living with the father of the children or the use of oral contraceptives or LARCs, beyond the risk factors present for first conception. However, we deduced from the qualitative evidence that risk factors and reasons for repeat unintended pregnancy appeared diverse and included:

*Contextual factors,* such as lack of family or peer support, educational or vocational opportunities, and chaotic lifestyles [48, 51, 53],

*“…we just went and did it” … “it was a spur of the moment thing… we were partying.”* (Herrman 2006, USA, teen mothers recruited from social service agencies)

*Emotional factors*, particularly to fill an emotional void after an abortion or adoption [49, 52],

“*I was just devastated carrying a baby for nine months and feeling it move, going through labour and everything and seeing him for the first time and him just going. It was horrible. He went to foster carers within days after birth.”* (Clarke 2010, UK and Caribbean, adolescents with two or more pregnancies in London)

*Practical factors,* such as the desire to complete one’s family whilst still young [48, 49, 53, 62],

For example, one teen said, *“My baby needs a brother or sister—it is too sad to see him growing up without someone to play with.”* Another mother stated, *“Now that I've had one, I should just finish it, you know, before going back to school and dropping out all over again.”* (Bull 1998, USA, teen mothers who received state food aid, and their mothers/guardians)

*Motivational factors*, such as personal goals and aspirations prompted young women to attempt to avoid a repeat pregnancy, but they were often not given the appropriate support to achieve their goals [49, 52, 63],

*“Creche facilities to allow you to go and finish your education and go out and get a job, then you are off the social. Why don't they do things like that?”* (Clarke 2010, UK and Caribbean, adolescents with two or more pregnancies in London)

## Discussion

Our multi-stream review aimed to understand different aspects of the existing literature. We found no quantitative evidence for the effectiveness of any intervention. The interventional studies reported psychosocial programmes conducted via home visits, community interventions or over the telephone. Meta-analyses found no statistically significant reduction in repeat pregnancies, although there was a reduction in live births. The qualitative data could not illuminate the reasons why these interventions did not produce significant improvements in the repeat unintended conception rate, but it helped to explain the context in which repeat pregnancies took place, and it offered an insight into young women’s lives, where choices were restricted, support was limited and opportunities were scarce. The realist component applied realist principles to appraise the evidence and provided a conceptual platform highlighting multiple mechanisms (e.g. connectedness and tailoring) that interacted with context and, if attended to, could increase the likelihood of an intervention being effective in preventing rapid repeat adolescent pregnancy.

### Who is at greatest risk of repeat unintended pregnancies?

There is clear set of risk factors associated with first pregnancies in young women, which include low socioeconomic status, being a care leaver, having low educational attainment and being the victim of abuse [37, 38, 46]. However, the majority of quantitative studies that examined the risk factors associated with repeat pregnancies were judged to be of poor quality with inconclusive findings; hence, they made no useful contribution to the review.

The qualitative evidence did explore the perceptions of young mothers’ reasons behind repeat pregnancies in young women. The explanations for repeat pregnancies range from contextual, motivational and emotional or vary according to the adolescent mother’s own rationale (i.e. whether a pregnancy is intended or not). It is important to seek to understand these complex and diverse reasons that result in some young mothers having multiple pregnancies so that measures can be developed to address individual issues through targeted, personalised interventions and improve provision of services.

### Which interventions are effective and cost-effective, how do they work, in what setting and for whom?

The trial interventions provided no significant evidence of effectiveness for either psychosocial interventions or simply improving access to contraceptive use on reducing repeat pregnancies. After including further sources of effectiveness evidence in a sensitivity analysis, we found that psychosocial visits delivered in a home-based setting reached statistically significant effectiveness. This tentative quantitative finding was very much supported by the evidence and feedback from both stakeholders and service users, who concluded that the home setting felt personal and provided more opportunity to discuss things that could not be discussed in a group setting. Home visits also had less of an impact on practical preparation time for the mothers with regard to organising themselves and their child or children to attend groups. Professionals suggested that home visits are more likely to be useful than interventions that rely on young women travelling to a clinic.

There was almost a compete dearth of economic and cost evaluation in the included studies. Despite the Barnet study showing no significant effectiveness on adolescent conception rates, the study provided some preliminary data on costs [10].

### What are the barriers to and facilitators of the uptake of these interventions and their ultimate success in reducing repeat adolescent pregnancies?

There are several possible reasons for the fact that we found no successful interventions for repeat pregnancy. A lack of high-quality, well-powered research is a clear factor; however, a key barrier may be the successful implementation of the intervention. The pressures and influences facing young adults shape their views, experience and negotiation of relationships and motherhood. These factors motivate them either to take control and protect against pregnancy, or to take a more relaxed approach to these issues. The views of young mothers during the service user feedback highlighted the importance of tailoring interventions within this broader context, with an appreciation of the multiple roles that an adolescent mother has to play, which include student, employee, friend and daughter. It is important to engage young women with this issue. It needs to be clear to them that they are being heard and that the choice to have safe sex is theirs. This gives them a clear perception of control of their bodies, decisions and lives. The evidence base has highlighted that context, motivation, planning for the future, taking control, situating the intervention within a broad context, connectedness and tailoring provide a conceptual framework to assist in guiding future research.

There is inconsistent evidence reported by previous systematic reviews on intervention programmes aimed at reducing repeat adolescent pregnancies. Furey [64] conducted a review citing two programmes [43, 44] that claimed to be successful in reducing the incidence of repeat pregnancy. However, careful examination of the data in these studies did not find a statistically significant reduction in the incidence. Corcoran and Pillai [65] performed a meta-analysis to investigate the effect of adolescent parent programmes on reducing repeat adolescent pregnancy; this analysis showed a reduction in the incidence of repeat pregnancy at follow-up on average 19 months after the intervention and also further reduction by second follow-up at 31 months. The current review showed dissipation after 24 months. This disparity between the findings of the current and previous review arose because there were slightly different inclusion criteria in this review and because we applied rigorous statistical methods for the analysis. Although some published literature reviews exist for programmes trialled within the USA, there are no systematic reviews of the evidence that address programmes to reduce the incidence of repeat adolescent pregnancy and the risk factors or reviews that scrutinise the reasons behind the success or failure of these programmes [66–68].

The strengths of our review include extensive literature searches and the use of a mixed-method streamed approach to address the multi-perspective aims of the review. The perspectives of service providers and service users were integrated with the findings from the literature. The main limitations of our review are that the included studies rarely characterised conceptions and pregnancies in young women and girls as ‘unintended’ or as ‘planned’. The interventions were often in place to provide alternatives to second pregnancies through programmes of empowerment, education and social contact, but without regard to intention.

#### Implications for research

The paucity of well-conducted research in this area, together with a lack of good candidate interventions, indicates that there is still considerable scope for investigating both methods for reducing repeat unintended conceptions in young women and for evidencing their effectiveness. It is important to consider the views of young people and to design interventions to address their motivations and beliefs as well as their practical needs. There is a necessity for more research on hard-to-reach groups who may be particularly vulnerable to repeat adolescent conceptions; however, it was not possible to clearly identify such groups in this review because of the lack of evidence. These groups could include looked-after children, drug or alcohol users, sex workers, homeless young people, asylum seekers and those caught up in the justice system. The likelihood of conducting randomised trials may be low in these subgroups because of the difficulties inherent in identifying, recruiting and retaining such young people in studies; therefore, high-quality qualitative research is recommended.

#### Implications for clinicians and policy makers

Whilst adolescent conceptions rates in the UK and elsewhere in Europe have fallen over recent years, the challenge and health impact of adolescent pregnancy first pregnancy in young women remains significant. We found a paucity of well-conducted research in this area and a lack of good candidate interventions. The realist findings indicate that new interventions need to include the perspectives of young mothers and to emphasise mechanisms such as connectedness and tailoring. Clinicians and policy makers need to be aware of barriers experienced by service users. These could be physical barriers, such as difficulty in getting to services through transport difficulties, to psychological barriers of feeling judged by health-care professionals or reception staff when booking appointments. The effectiveness and cost-effectiveness of new interventions need to be tested in suitably powered RCTs and concurrent economic evaluations.

## Conclusions

No conclusive evidence for the effectiveness of any interventions to reduce repeat pregnancy in young mothers was identified. However, ‘the absence of evidence is not evidence of absence’.

There was some, weak quantitative evidence indicating that home-delivered, multi-component, complex psychosocial interventions may be effective in reducing conceptions in young mothers and subsequent births, and may help young mothers to remain in education. This evidence was strengthened and supported by the qualitative evidence and realist synthesis. More rigorously conducted and better-reported studies are needed, and the other goals of adolescent parenting programmes, beyond simple reduction in the incidence of pregnancy, need to be subjected to rigorous quantitative scrutiny.

## Additional file

Additional file 1:
**Section 1:** Search strategies. **Section 2:** Studies used in meta-regression and their references. **Section 3:** Summary of quantitative studies. **Section 4:** Summary of qualitative studies. **Section 5:** Risk of bias summary: review authors' judgements about each risk of bias item for each included study. **Section 6:** Summary of quantitative findings — GRADE profile. **Section 7:** Summary of qualitative findings — CERQual profile. **Section 8:** Sensitivity analysis forest plots. **Section 9:** Realist Summary statements of emerging theory areas. **Section 10:** Overarching synthesis table. (DOCX 721 kb)

## Abbreviations

AIDS: Acquired immunodeficiency syndrome
ASSIA: Applied Social Sciences Index and Abstracts
BiblioMap: The EPPI-Centre register of health promotion and public health research
BNI: British Nursing Index
CAMI: Computer-Assisted Motivational Interviewing
CAMI+: Computer-Assisted Motivational Interviewing with a multi-part home visit programme
CART: Completeness, Accuracy, Relevance and Timeliness
CBA: Cost benefit analysis
CERQual: Confidence in the Evidence from Reviews of Qualitative research
CI: Confidence interval
CINAHL: Cumulative Index to Nursing and Allied Health Literature
CONSORT: Consolidated Standards of Reporting Trials
EconLit: American Economic Association’s electronic bibliography
EIP: Early Intervention Program
Embase: Excerpta Medica database
EPOC: Effective practice and organisation of care
EPPI-Centre: Evidence for Policy and Practice Information and Co-ordinating Centre
ERIC: Educational Resource Index and Abstracts
GRADE: Grading of Recommendations Assessment, Development and Evaluation
HIV: Human immunodeficiency virus
ICER: Incremental cost-effectiveness ratio
INTERUPT: Intervention Now To Eliminate Repeat Unintended Pregnancy in Teenagers
ITS: Interrupted time series
IUCD: Intra-uterine contraceptive device
IUD: Intrauterine device
LARC: Long acting reversible contraceptive
MMAT: Mixed-Methods Appraisal Tool
MOOSE: Meta-Analysis and Systematic Reviews of Observational Studies
NHS: National Health Service
NWORTH: North Wales Organisation for Randomised Trials in Health
PHW: Public Health Wales
PICO: Patient Intervention Comparison Outcome
PRISMA: Preferred Reporting Items for Systematic Reviews and Meta-Analyses
PROGRESS: Place, Race, Occupation, Gender, Religion, Education, Socioeconomic status (SES), Social status
RAMESES: Realist And Meta-narrative Evidence Syntheses: Evolving Standards
RCT: Randomised control trial
RePEc: Research Papers in Economics
RoB: Risk of bias
RR: Risk ratio
SD: Standard deviation
SES: Socioeconomic status
SocAbs: Sociological Abstracts
STD: Sexually transmitted disease
SURE: Support Unit for Research Evidence
TOP: Termination of pregnancy
UN: United Nations
