# Supplementary material for: Intervention Now to Eliminate Repeat Unintended Pregnancy in Teenagers (INTERUPT): a systematic review of intervention effectiveness and cost-effectiveness, and qualitative and realist synthesis of implementation factors and user engagement
Source: BMC Med. 2017 Aug 15;15:155. doi: 10.1186/s12916-017-0904-7 (PMC5557469; doi:10.1186/s12916-017-0904-7)
Supplement: Additional file 1: — Section 1: Search strategies. Section 2: Studies used in meta-regression and their references. Section 3: Summary of quantitative studies. Section 4: Summary of qualitative studies. Section 5: Risk of bias summary: review authors' judgements about each risk of bias item for each included study. Section 6: Summary of quantitative findings — GRADE profile. Section 7: Summary of qualitative findings — CERQual profile. Section 8: Sensitivity analysis forest plots. Section 9: Realist Summary statements of emerging theory areas. Section 10: Overarching synthesis table. (DOCX 721 kb) [file 12916_2017_904_MOESM1_ESM.docx]

# Section 1, Search strategies

**Medline**

1. exp pregnancy in adolescence/
2. Pregnancy, unplanned/
3. Pregnancy unwanted/
4. Abortion, induced/
5. Abortion, legal/
6. Or/2-5
7. Adolescent/
8. (Adolescen$ or teen$ or girl or girls or youth or youths or youthful or young or juvenile).ti,ab.
9. 7 or 8
10. (Repeat or subsequent or second or secondary or further or additional).ti,ab.
11. 1 and 10
12. 6 and 9 and 10
13. 11 or 12
14. Limit 13 to 1995 onwards and humans only

**Hand searches**

Journals were identified by comparing those that published RCTs and qualitative studies included in the review with a Cochrane register of journals and selecting those English language journals not including in the register.

1. Family relations
2. Journal of perinatal education
3. Wisconsin medical journal
4. Annals family med
5. Matern child health j
6. Journal of family issues
7. Health care forwomen international
8. Journal of psychosocial nursing,
9. Midris midwifery digest
10. Community practitioner
11. Journal of adolescent health
12. International quarterly of community health education
13. Nursing connect
14. Journal of health care for the poor and underserved
15. Children & society
16. Early child development and care
17. Perspect sex reprod health

The electronic indexes of the above journals were screened for relevant titles from January 2010.

#

# Section 2, Studies used in meta-regression and their references

| **Author, Year** | **EPOC** |
| --- | --- |
| Berenson, 1997 | Non-comparative study |
| Coard, 2000 | Non-comparative study |
| Crittenden, 2009 | Non-comparative study |
| Drayton 2000, Drayton et al 2002a and 2002b | Non-comparative study |
| Kershaw, 2003 | Non-comparative study |
| Lewis, 2010 | Non-comparative study |
| Mbambo, 2006 | Non-comparative study |
| Omar, 2008 | Non-comparative study |
| Persona, 2004 | Non-comparative study |
| Salihu, 2011 | Non-comparative study |
| Stevens-Simon, 1995 | Non-comparative study |
| Stevens-Simon, 1998 | Non-comparative study |
| Stevens-Simon, 1999 | Non-comparative study |
| Stevens-Simon, 2001 | Non-comparative study |
| Wang, 2005 | Non-comparative study |
| Williams, 2001 | Non-comparative study |
| Mulsow, 1996 | Prospective cohort study |
| Ranieri, 2007 | Prospective cohort study |
| Sant'Anna, 2007 | Prospective cohort study |
| Templeman, 2000 | Prospective cohort study |
| Thurman, 2007 | Prospective cohort study |
| Mears, 1997 | Prospective cohort study |
| Bruno, 2009 | Prospective cohort study |
| Falk, 2006 | Retrospective cohort study |
| Key, 2001 | Retrospective cohort study |
| O'Dell, 1998 | Retrospective cohort study |
| Patchen, 2009 | Retrospective cohort study |
| Paukku, 2003 | Retrospective cohort study |
| Richio, 2010 | Retrospective cohort study |
| Sangalang, 2006 | Retrospective cohort study |
| Barnet, 2008 | Retrospective cohort study |

*Prospective cohort studies*

Bruno Z, Feitosa F, Silveira K, de Morais I, Bezerra M. Subsequent pregnancy among adolescents. [Portuguese] Reincidencia de gravidez em adolescentes. Revista Brasileira de Ginecologia e Obstetricia 2009;31(10):480-84.

Cavazos-Rehg PA, Krauss MJ, Spitznagel EL, Schootman M, Cottler LB, Bierut LJ. Associations Between Multiple Pregnancies and Health Risk Behaviors Among U.S. Adolescents. Journal of Adolescent Health 2010;47(6):600-03.

Key JD, Gebregziabher MG, Marsh LD, KM OR. Effectiveness of an Intensive, School-Based Intervention for Teen Mothers. J Adolesc Health 2008;42:394-400.

Lewis CM, Faulkner M, Scarborough M, Berkeley B. Preventing Subsequent Births for Low-Income Adolescent Mothers: An Exploratory Investigation of Mediating Factors in Intensive Case Management. American Journal of Public Health 2012;102(10):1862-65.

Mears C, Hediger M, Martin S, Scholl T, Kramer J. Social factors prediciting postpartum choice of Norplant among African American and non-Hispanic white adolescents. Journal of Adolescent Health 1997;21:167-71.

Mulsow MH, Murry VM. Parenting on edge: economically stressed, single, African American adolescent mothers. Journal of Family Issues 1996;17(5):704-21.

Raneri LG, Wiemann CM. Social ecological predictors of repeat adolescent pregnancy. Perspectives on Sexual and Reproductive Health 2007;39(1):39-47.

Rubin V, East P. Adolescents' Pregnancy Intentions: Relations to Life Situations and Caretaking Behaviors Prenatally and 2 Years Postpartum. Journal of Adolescent Health 1999;24:313-20.

Sant'Anna MJC, Carvalho KAM, Melhado A, Coates V, Omar HA. Teenage pregnancy: Impact of the integral attention given to the pregnant teenager and adolescent mother as a protective factor for repeat pregnancy. The Scientific World Journal 2007;7:187-94.

Silva ADA, Coutinho IC, Katz L, Souza ASR. A case-control study of factors associated with repeat teen pregnancy based on a sample from a university maternity hospital. Cad. Saude Publica 2013;29(3):496-506.

Stevens-Simon 1995, 1998

Stevens-Simon C, Wallis J, Allen-Davis J. Which teen mothers choose Norplant? . Journal of Adolescent Health, 1995; 16(5):350-35

Stevens-Simon C and K. L, Correlates and consequences of early removal of Levonorgestrel implants among teenaged mothers. Arch Pediatr Adolesc Med. , 1998a. 152: p. 893-898.

Stevens-Simon C, Kelly L, Singer D. Preventing repeat adolescent pregnancies with early adoption of the contraceptive implant. . Family Planning Perspectives 1999;31(2):88-93.

Teal SB, Sheeder J. IUD use in adolescent mothers: retention, failure and reasons for discontinuation. Contraception 2012;85(3):270-74.

Templeman CL, Cook V, Goldsmith LJ, Powell J, Hertweck SP. Postpartum contraceptive use among adolescent mothers. Obstet Gynecol 2000;95(5):770-6.

Thurman AR, Hammond N, Brown HE, Roddy ME. Preventing Repeat Teen Pregnancy: Postpartum Depot Medroxyprogesterone Acetate, Oral Contraceptive Pills, or the Patch? Journal of Pediatric and Adolescent Gynecology 2007;20(2):61-65.

*Retrospective cohort studies*

Falk G, Ostlund I, Magnuson A, Schollin J, Nilsson K. Teenage mothers - a high-risk group for new unintended pregnancies. Contraception 2006;74(6):471-75.

Key JD, Barbosa GA, VJ O. The Second Chance Club: Repeat adolescent pregnancy prevention with a school-based intervention. . Journal of Adolescent Health 2001;28(3):167-69.

Key JD, O'Rourke K, Judy N, A MS. Efficacy of a secondary adolescent pregnancy prevention program: An ecological study before, during and after implementation of the second chance club. International Quarterly of Community Health Education 2006;24(3):231-40.

O'Dell C. M., Forke C. M., Polaneczky M. M., Sondheimer S. J., Slap G. B. Depot medroxyprogesterone acetate or oral contraception in postpartum adolescents. Obstet. Gynecol. 1998;91(4):609-14.

Patchen L, Caruso D, Lanzi RG. Poor maternal mental health and trauma as risk factors for a short interpregnancy interval among adolescent mothers. Journal of Psychiatric and Mental Health Nursing 2009;16(4):401-03.

Paukku M, Quan J, Darney P, Raine T. Adolescents' contraceptive use and pregnancy history: is there a pattern? Obstet Gynecol 2003;101(3):534-8.

Richio LJ, Phipps MG, Raker CA. Repeat teen birth: Does delivery mode make a difference? American Journal of Obstetrics and Gynecology 2010;203(5):453.e1-53.e5.

Sangalang BB, Barth RP, Painter JS. First-Birth Outcomes and Timing of Second Births: A Statewide Case Management Program for Adolescent Mothers. Health & Social Work 2006;31(1):54-63.

*Non comparison studies*

Berenson A, Wiemann C. Contraceptive use among adolescent mothers at 6 months postpartum. Obstet. Gynecol. 1997;89(6):999-1005.

Carvajal D, Burrell L, Duggan A, Barnet B. Repeat pregnancy prevention self-efficacy in adolescents: Associations with provider communication, provider type, and depression. Southern Medical Journal 2012;105(11):591-97.

Coard SI, Nitz K, Felice ME. Repeat Pregnancy among Urban Adolescents: Sociodemographic, Family, and Health Factors. Adolescence 2000;35(137):193-200.

Collins ME, Stevens JW, Lane TS. Teenage parents and welfare reform: findings from a survey of teenagers affected by living requirements. Social Work 2000;45(4):327-38.

Crittenden CP, Boris NW, Rice JC, Taylor CA, Olds DL. The role of mental health factors, behavioral factors, and past experiences in the prediction of rapid repeat pregnancy in adolescence. Journal of Adolescent Health 2009;44(1):25-32.

da Silva KS, Rozenberg R, Bonan C, Chuva VCC, da Costa SF, Gomes M. Repeated pregnancy among adolescents and social vulnerability in Rio de Janeiro (RJ, Brazil): data analysis of Information System on Live Births. Cienc. Saude Coletiva 2011;16(5):2485-93.

Drayton 2000; 2002a; 2002b.

Drayton VLC, Montgomery SB et al. The impact of the Women's Centre of Jamaica Foundation programme for adolescent mothers on repeat pregnancies. The West Indian Medical Journal. 2000; 49(4): 316-326.

Drayton VLC. Contraceptive use among Jamaican teenage mothers. Revista Panamericana de Salud Publica/Pan American Journal of Public Health. 2002a; 11(3): 150-157.

Drayton VLC, Montgomery SB et al. The Health Belief Model as a predictor of repeat pregnancies among Jamaican teenage mothers. International Quarterly of Community Health Education. 2002b; 21(1): 67-81. 2000.

Flynn L. The Adolescent Parenting Program: Improving outcomes through mentorship. Public Health Nursing 1999;16(3):182-89.

Gray S, Sheeder J, O'Brien R, Stevens-Simon C. Having the best intentions is necessary but not sufficient: What would increase the efficacy of home visiting for preventing second teen pregnancies? Prevention Science 2006;7(4):389-95.

Kershaw TS, Niccolai LM, Ickovics JR, Lewis JB, Meade CS, Ethier KA. Short and long-term impact of adolescent pregnancy on postpartum contraceptive use: implications for prevention of repeat pregnancy. Journal of Adolescent Health 2003;33(5):359-68.

Lewis L. N. 2010a; 2010b.

Lewis LN, Doherty DA et al. Predictors of sexual intercourse and rapid-repeat pregnancy among teenage mothers: An Australian prospective longitudinal study. Medical Journal of Australia 2010;193(6):338-342

Lewis LN, Doherty DA et al. Implanon as a contraceptive choice for teenage mothers: a comparison of contraceptive choices, acceptability and repeat pregnancy. Contraception 2010;81:421-426.

Mapanga KG, Andrews CM. The influence of family and friends' basic conditioning factors and self-care agency on unmarried teenage primiparas' enagagement in contraceptive practice. J Community Health Nurs 1995;12(2):89-100.

Mbambo DE, Ehlers VJ, Monareng LV. Factors influencing adolescent mothers' non-utilisation of contraceptives in the Mkhondo area. Health SA Gesondheid 2006;11(4):22-31.

Omar H. A., Fowler A., McClanahan K. K. Significant Reduction of Repeat Teen Pregnancy in a Comprehensive Young Parent Program. Journal of Pediatric and Adolescent Gynecology 2008;21(5):283-87.

Padin M. D. R., Silva R. D. E., Mitsuhiro S. S., Chalem E., Barros M. M., Guinsburg R., et al. Repeat pregnancies among adolescents in a tertiary hospital in Brazil. J. Reprod. Infant Psychol. 2012;30(2):193-200.

Persona L, Shimo AK, Tarallo MC. Profile of adolescents with repeated pregnancies attended at a prenatal clinic. [Portuguese] Perfil de adolescentes com repeticao da gravidez atendidas num ambulatorio de pre-natal. Revista Latino-Americana de Enfermagem 2004;12(5):745-50.

Sadler LS, Swartz MK, Ryan-Krause P, Seitz V, Meadows-Oliver M, Grey M, et al. Promising Outcomes in Teen Mothers Enrolled in a School-Based Parent Support Program and Child Care Center. Journal of School Health 2007;77(3):121-30.

Salihu HM, August EM, Jeffers DF, Mbah AK, Alio AP, Berry E. Effectiveness of a Federal Healthy Start Program in Reducing Primary and Repeat Teen Pregnancies: Our Experience over the Decade. Journal of Pediatric and Adolescent Gynecology 2011;24(3):153-60.

Schaffer MA, Goodhue A, Stennes K, Lanigan C. Evaluation of a public health nurse visiting program for pregnant and parenting teens. Public Health Nursing 2012;29(3):218-31.

Stevens-Simon C, Kelly L, D S. Absence of negative attitudes towards childbearing among pregnant teenagers. Arch Pediatr Adolesc Med. 1996;150:1037-43.

Stevens-Simon C, Kelly L, R K. A village would be nice but...It takes a long-acting contraceptive to prevent repeat adolescent pregnancies. American Journal of Preventive Medicine 2001;21(1):60-65.

Stevens-Simon C, Kelly L, Singer D, D. N. Reasons for first teen pregnancies predict the rate of subsequent teen conceptions. Pediatrics 1998b;101:e8.

Swedish KA, Rothenberg A, Fuchs K, Rosenberg G. Successful life navigation by former participants in a group for pregnant and parenting teens. Vulnerable Children and Youth Studies 2010;5(4):310-21.

Truong HHM, Kellogg T, McFarland W, Kang MS, Darney P, Drey EA. Contraceptive intentions among adolescents after abortion. Journal of Adolescent Health 2006;39(2):283-86.

Viellas E.F., Granado Nogueira da Gama S., Theme Filha M.M., do Carmo Leal M. Repeated pregnancy among adolescents and negative outcomes of the newborn: study in the city of Rio de Janeiro.. Rev Bras Epidemiol 2012;15(3):443-54.

Wang R, Wang H. Prebirth psychosocial factors as predictors of consistency in contraceptive use among Taiwanese adolescent mothers at 6 months postpartum. Public Health Nursing 2005;22(4):271-79.

Williams EG, Sadler LS. Effects of an Urban High School-Based Child Care Center on Self-Selected Adolescent Parents and Their Children. Journal of School Health 2001;71(2):47-52.

Wilson EK, Fowler CI, Koo HP. Postpartum Contraceptive Use Among Adolescent Mothers in Seven States. Journal of Adolescent Health 2013;52(3):278-83.

Patchen L, Letourneau K, Berggren E. Evaluation of an integrated services program to prevent subsequent pregnancy and birth among urban teen mothers. Soc Work Health Care 2013;52(7):642-55.

# Section 3, Summary of quantitative studies

| **Author/ Year / Country** | **Sample size and  PROGRESS** | **Type of Program and Length of Intervention** | **Program Description** | **Usual Care** | **Main narrative findings** |
| --- | --- | --- | --- | --- | --- |
| **Belzer, 2005 USA** | N = 160 (69 lost to follow-up; 91 analysed); intervention group, n = 43; control group, n = 48. Hispanic, n= 131; non- Hispanic (African American, Caucasian, Asian Pacific | Contraceptive; 10–15 minutes of education about emergency contraception plus unspecified amount of family planning services | Provision of emergency contraception was provided by a licensed health professional | 10-15 mins of education about emergency contraception | The advance provision of emergency contraception in young mothers increases the likelihood of its use, and does not affect the use of other contraceptive methods like condoms, or hormonal methods of birth control. |
| **Barnet, 2009 USA** | N = 235 (predominantly African American); CAMI+, n = 80; CAMI only, n = 87; control, n= 68 | Psychological, home visit:  CAMI sessions were  initiated by 6 weeks  postpartum and  continued quarterly  through 24 months  postpartum | Delivered by African American  counsellors. (1) CAMI+: home  visiting with CAMI; training  through a 16-module  curriculum specifically for urban  African-American adolescent  mothers which addressed  age- and developmentally  appropriate feeding, growth,  play and discipline; three  modules focused on safer sex,  negotiation and goal-setting.  (2) CAMI only: single  component intervention  motivational interviewing | usual care | Receipt of two or more CAMI  sessions, either alone or within  a multicomponent home-based  intervention, reduced the risk  of rapid subsequent birth to  adolescent mothers (not  statistically significant) |
| **Black, 2006 USA** | Initially recruited to  intervention, n= 87;  control, n= 94. African  American with low-income  SES | Psychosocial, home visit:  received home visits every  other week until the  infant’s first birthday, for  a maximum of 19 visits | Intervention delivered by  college-educated, black  single mothers who served  as mentors (‘big sisters’).  Curriculum focused on  interpersonal negotiation skills,  adolescent development and  parenting. Condoms were  provided at every contact | Initial information on services  for young mothers and  children | A home-based intervention  founded on a mentorship  model and targeted towards  adolescent development,  including negotiation skills,  was effective in preventing  rapid repeat. At the 2-year  evaluation, there were no other  differences in pregnancy rates  between intervention- and  control-group mothers |
| **Barnet, 2007  USA** | Home-visited group, n = 44;  control group, n = 40 | Psychosocial, home visit:  started in the third  trimester and then  biweekly for the first year  of the child’s life and then  monthly until the child’s  second birthday | Trained home visitors, recruited  from local communities,  delivered a parenting  curriculum, an adolescent  curriculum, encouraged  contraceptive use, connected  the teen with primary care and  promoted school continuation.  Training was also geared  towards improving  communication and negotiation  with partners. Visitors taught to  identify depression and partner  violence | Usual care | This community-based home visiting  programme improved  adolescent mothers’ parenting  attitudes and school  continuation, but it did not  reduce their odds of repeat  pregnancy or depression, or  achieve co-ordination with  primary care. Co-ordinated  care may require explicit  mechanisms to promote  communication between the  community programme and  primary care |
| **Stevens-Simon 1997 USA** | N = 286; place: USA.  44% white, 25% black,  29% Hispanic and  2% other races | Psychosocial - community | Four interventions:  (1) monetary incentive and  peer-support; (2) peer-support;  (3) monetary incentive; and  (4) no intervention. Peer-based  incentive programme ‘Dollar-a-  Day’: meet weekly, mentor-led  peer group environment; guest  speakers, free contraceptive  services and supplies and  information about jobs and  job-shadowing experiences are  available on-site | The control group received  three different versions of the  intervention: (1) peer-support;  (2) monetary incentive; and  (3) usual care | Adolescent mothers  participating in a weekly peer  support group meeting during  which they learn about the  advantages of delaying further  childbearing and the costs of  repeat pregnancy have fewer  second pregnancies during the  2 years following birth.  However, this was not  statistically significant |
| **Ford 2002 USA** | N = 282 (93.1% African  American); experimental,  n = 165 (95.7% African  American); control, n = 117  (89.4% African American).  Years of schooling (range):  8–14 | Psychosocial, community:  scheduled clinic time | Peer-centred prenatal care  programme for adolescent  mothers | Usual care and a box of  educational materials that  included written information  on the three trimesters of  pregnancy | Adolescents in the experimental  group had fewer infants with  low birthweight and were  more likely to continue their  education than those in the  control group. However, this  was not statistically significant |
| **Havens, 1997 USA** | N = 110; experimental,  n = 53 (3 white, 47 black, 2 Hispanic, 1 nativeAmerican); control, n = 57  (3 white, 53 black, 0 Hispanic, 1 native  American). Occupation:  students | Psychosocial, home visit:  minimum of 12 hours  each month with teens  over the 2-year period  of the project | Focus on telephone conversations and social  outings. Mentors were expected to develop  relationships with their teens  that fostered trust and  friendship then focus on  goal-setting: contraception,  continuing in school,  day-care, parenting and  budgeting and to deal with  young mothers problems | Assistance from the  community agency and social  support from family and  friends | The mentoring programme did  not significantly impact repeat  pregnancy rates |
| **Koniak-Griffin, 2003 USA** | Experimental group, n = 56;  control group, n = 45.  Latina, n = 63; African  American, n = 13;  non-Hispanic white,  n = 18; other, n = 4.  SES: ‘poor’ | Psychosocial, home visit:  gestation age of 28 weeks  or more from pregnancy  to 1 year postpartum.  The ‘Early Intervention  Program’ was designed to  include a maximum of  17 home visits: 2 prenatal  and 15 postpartum (1.5 to  2 hours each); however,  the number of visits  varied among families  depending on the  mother’s availability | Comprehensive interventions  were provided by public health  nurses in five major areas:  health, sexuality and family  planning, maternal role, life  skills, and social support.  Prenatal visits focused on  health care during pregnancy  and preparation for childbirth,  and maternal roles and mental  health issues. Postpartum visits  focused on family planning,  infant care, and well-baby  health care, demonstration of  the Neonatal Behaviour  Assessment Scale, educational  and vocational goals, and video  instruction for promoting  appropriate maternal  behaviours | The TPHNC mothers received  services available in county  health departments. One  prenatal visit was made shortly  after the participant’s entry  into the study and a second  during the third trimester.  Visits focused on  (1) assessment and counselling  related to prenatal health care,  (2) self-care, (3) preparation for  childbirth, (4) education  planning, and (5) well-baby  care, including immunizations | The lower repeat pregnancy  rate in adolescent mothers  who received home visitation,  although not statistically  significant, is clinically  important because of the  negative impact short-interval  births may have on the life  course of these mothers and  their children |
| **Quinlivan, 2003 Australia** | Experimental, n = 65  (21 indigenous Australians);  control group, n= 71  (12 indigenous Australians) | Psychosocial, home visit:  6 months postpartum  visits were undertaken  at 1 week, 2 weeks,  1 month, 2 months,  4 months and 6 months  after birth. Each visit  lasted 1–4 hours | Postnatal home visits by  midwives, routine postnatal  support, counselling and  information services provided  by the hospital, including  access to routine hospital  support services: teach  breastfeeding and maternal  infant bonding skills; provide  advice and information on  contraception and on  vaccination of infant; discuss  postnatal mood disorders and  mother’s feelings; follow up  abnormalities detected in the  antenatal period, such as pap  smear problems, infectious  diseases, substance misuse or  family violence issues; ensure  appointments are made for  vaccination of infant; provide  training in parenting skills,  covering issues that will emerge  with the increasing mobility of  the child, and talk about child  safety in the home | All participants were provided  with routine postnatal support,  counselling and information  services provided by the  hospital, including access to  routine hospital support  services | Postnatal home-visiting services  by nurse midwives reduce  adverse neonatal events and  improve contraception  outcomes, but do not affect  breastfeeding or infant  vaccination knowledge or  compliance |
| **Katz, 2011 USA** | N = 249 (221 African  American, 28 Latina);  intervention group, n= 124;  usual care, n = 125. Place:  USA. SES: 166 on Medicaid  48 undertaking non-schoolbased  job-training and  126 held back in school | All of the family  advocates were female  and African American.  Biweekly phone sessions  were scheduled during  the subsequent  12 months with a  maximum projected  number of 42 phone  counselling sessions  over an 18-month  postpartum interval | Counsellors were Masters-level  young women of similar ethnic  backgrounds as the teens.  Curriculum content focused on  building knowledge of health  risks and developing positive  teen attitudes and skills for  future orientation and  self-regulation. Other issues  addressed included improving  communication and negotiation  skills with sexual partner,  resisting peer pressures for risk  behaviours, and increasing  connectedness with family,  health provider, school and  work settings. Minimal content  addressed parenting or child  development. A workbook with  visual support materials for the  topics slated for discussion.  Two-hour dinner group  sessions at one of the centrally  located hospitals serving  pregnant and parenting teens | Participants randomised to  usual care received the health  and education services  generally provided through  their schools or health-care  facilities | GirlTalk intervention did not  reduce the time before a  subsequent pregnancy for the  15–19-year-old sample as a  whole. Among adolescent  mothers aged 15–17 years, the  rate of subsequent pregnancy  was 26% in the intervention  group and 39% in the usual  care group. Subsequent  analysis suggested that  increased participation in the  GirlTalk counselling programme  was associated with postponing  a second pregnancy among  teen mothers aged 15–17 years,  but not those ≥18 years |
| **Sims, 2002 USA** | Experimental group, n = 48;  standard programme,  n = 51. 64% of the teens  who participated in the  24-month assessment were  African American, 29%  were European American,  3% were Hispanic and 4%  indicated that they were of  mixed race | Psychosocial, home:  designed for home  visitation 6 months  postpartum | The family support programme  involved weekly home visits  from a paraprofessional family  advocate. The family advocate  attempted to establish a  trusting relationship with the  teens and their families,  provided information about  services available in the  community (e.g. family  planning) and the care of  children, provided emotional  and instrumental support  (e.g. transportation), and  encouraged young mothers to  pursue the goals of the  programme (e.g. high school  completion and limiting further  childbearing), which included  helping the young mothers to  return to school and addressing  the psychological well-being of  the adolescents. needed in the  community, provided emotional  and instrumental support, and  encouraged the teens to  complete school | In the standard programme,  an advocate was housed at the  health centre and worked with  the teens almost exclusively via  the phone and mail. Contact  was less intensive and focused  on crisis management, thus  the rates of subsequent  pregnancies and births were  expected to be higher in the  standard programme group.  All the family advocates were  female, African American,  had at least a high school  education when the  programme began, and were  from the community where  the intervention took place | The two treatment groups did  not differ in their rates of  second pregnancies and second  births. The repeat pregnancy  rate was 58% for the homevisited  group and 63% for the  standard programme group.  The rates of second births were  31% and 35%, respectively |
| **Cherniss, 1996 USA** | N = 116 (50% African  Americans; 27% Latina;  17% white/non-Latina).  Place: USA; SES: low | Psychosocial – home-based family therapy. No set time  limit for treatment | Intensive family-based therapy  home visits, developmental  guidance, supportive  counselling, and help in  acquiring other needed  services. The family therapy  clients received these services,  the home-based family therapy  intervention | Individual treatment group  received home visits,  developmental guidance,  supportive counselling and  help in acquiring other needed  services . | The intervention with the  family improved the caring  environment for the infant  during the first 12 months of  treatment |

# Section 4, Summary of qualitative studies

| **Study, location** | **Data collection method and date** | **Participant details** | **Setting (including intervention if any)** | **Analysis method** | **Study focus/research question** |
| --- | --- | --- | --- | --- | --- |
| Bull, 1998  USA  3 urban and 2 rural counties, Georgia | Focus groups  Recorded and transcribed verbatim  Date not reported | 40 teen mothers, aged 16-19  29 African American, 9 white, 2 Hispanic  24 Mothers/guardians (1 male)  18 African American, 6 White | A state supplemental nutritional programme for women, infants and children that served >70% of teen mothers in Georgia | Content analysis using grounded theory and Tally text-based software for thematic coding | Factors associated with repeat pregnancy including pregnancy plans, contraceptive use, education, aspirations, relationships between teen mothers and their parents and the fathers of their children. Influence of the nutrition programme and other social service agencies |
| Clarke, 2010  Clarke, 2002 (thesis)  UK & Caribbean  London, Barbados and Jamaica | Interviews  Recorded and transcribed verbatim  Date not reported | 52 teens who had had 2 or more pregnancies  *London*  26 teens, aged 16-20  11 white, 10 Black, 3 Asian and 3 mixed race^[[1]](#footnote-1)^  9 no formal exam passes  19 in receipt of benefits  11 living with a partner  *Barbados & Jamaica*  26 ethnic Caribbean teens, aged 15-19  22 no formal exam passes  21 financial reliance on family and putative fathers  4 living with a partner | Ante natal clinics, statutory and voluntary social service agencies in 14 London boroughs  Social work department of major hospital in Barbados  Ante natal clinics of 2 public hospitals, and 2 poly clinics in Kingston, Jamaica | Framework analysis | To explore the psychosocial, emotional and economic factors which lead to repeat pregnancies |
| Hellerstedt, 1998  USA  Minnesota | Focus groups  Recorded and transcribed verbatim  Date not reported | 22 teen mothers, aged 13-19  7 African American, 6 Asian. 7 White, 2 Other  6 living with a partner  6 had been pregnant > once  All enrolled in secondary school  19 received Depo-Provera post delivery | All participants received health care at an agency serving a low income population, and with specific expertise in pregnant and parenting adolescents. | Thematic analysis | Decision making about contraceptive use and factors that would influence contraceptive discontinuation, particularly side effects of Depo-Provera |
| Herrman, 2006,  Herrman, 2007  USA  Urban location, city not named | Interviews  Recorded and transcribed verbatim  Date not reported | 16 teen mothers, aged 16-19  15 African American, 1 Hispanic  2 pregnant first time  14 pregnant > once | Recruited from social service agencies | Coding of data using Ethnograph 5.0 software and thematic analysis | Intentions and decision making associated with repeat pregnancy |
| Hoggart 2010  UK  London | Interviews, focus groups and discussion groups  Method of recording the data not reported  Date not reported | 23 teenage pregnancy strategy coordinators  14 providers of abortion referrals  8 abortion providers  16 school-based focus groups of 8-10 female participants aged 14-16  10 women aged 16-19 who had terminated pregnancies | 10 local health authorities in London  Schools in the study area.  7 of the young women who had had abortions were recruited from the British Pregnancy Advisory Service | Coding of data using NVivo software and thematic analysis | Teenage sexual behaviour and becoming pregnant, abortion decision-making and repeat abortion |
| Lewis, 2012  USA  Chicago | Interviews and follow up surveys at 6 and 12 months  Recorded and transcribed  October 2006 – August 2008 | 40 teen mothers, aged 15-18  All African American  All primiparous, ≤13 weeks postpartum  24 in relationships | The Postpartum Adolescent Birth Control Study. A longitudinal mixed methods study of African American adolescent mothers’ contraceptive use and risk of repeat pregnancy in the postpartum year | Coding of data using ATLAS.ti 5.0 software and thematic analysis | Partners’ roles in the use of non-coital dependent contraceptive methods (oral contraceptives, IUDs and Depo-Provera |
| Schaffer, 2008  USA  Minnesota  Urban location, city not named | Focus groups  Recorded and transcribed | 9 teen mothers, age not reported  5 African American, 2 Latino, 2 Caucasian | The Pregnancy Free Club. Delivered by Public Health Nurses and teachers in school, including monthly pregnancy tests, counselling, mentoring, contraception | Thematic analysis | Satisfaction with the experience of the Pregnancy Free Club |
| Smith, 2013  Australia  Perth | Interviews  Recorded and transcribed verbatim  May 2007 – January 2009 | 56 teens, aged 14-19  16 pregnant  19 had a baby  21 had recent terminations  8 Aboriginal  40 school dropouts  18 living with partner | Recruited from clinical and community-based antenatal and postnatal services | Additional thematic analyses were applied to a subset of data from a larger study which did not set out to explore pregnancy intentions but this theme inherently emerged in discussions | The relationship between pregnancy intentions and contraceptive behaviour |
| Weston, 2012  USA  Chicago | Interviews at baseline, 3, 6, 9 and 12 months postpartum  Recorded and transcribed verbatim | 20 teen mothers, aged 15-18  who expressed an intention to obtain the IUD in at least one interview  All African American | The Postpartum Adolescent Birth Control Study. A longitudinal mixed methods study of African American adolescent mothers’ contraceptive use and risk of repeat pregnancy in the postpartum year | Coding of data using ATLAS.ti 5.0 software and thematic analysis using a grounded theory approach | Barriers and facilitators to uptake of IUDs. |

# Section 5, Risk of bias summary: review authors' judgements about each risk of bias item for each included study


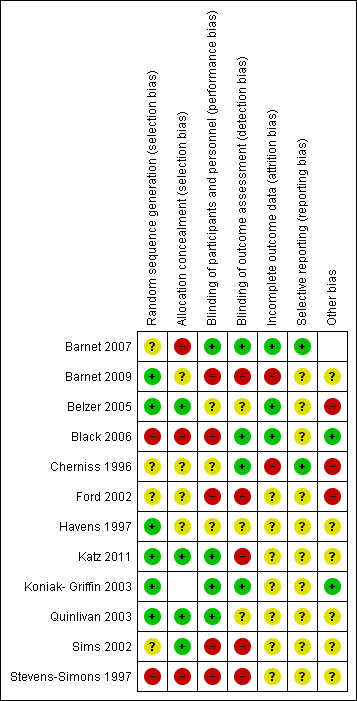


# Section 6, Summary of quantitative findings – GRADE profile


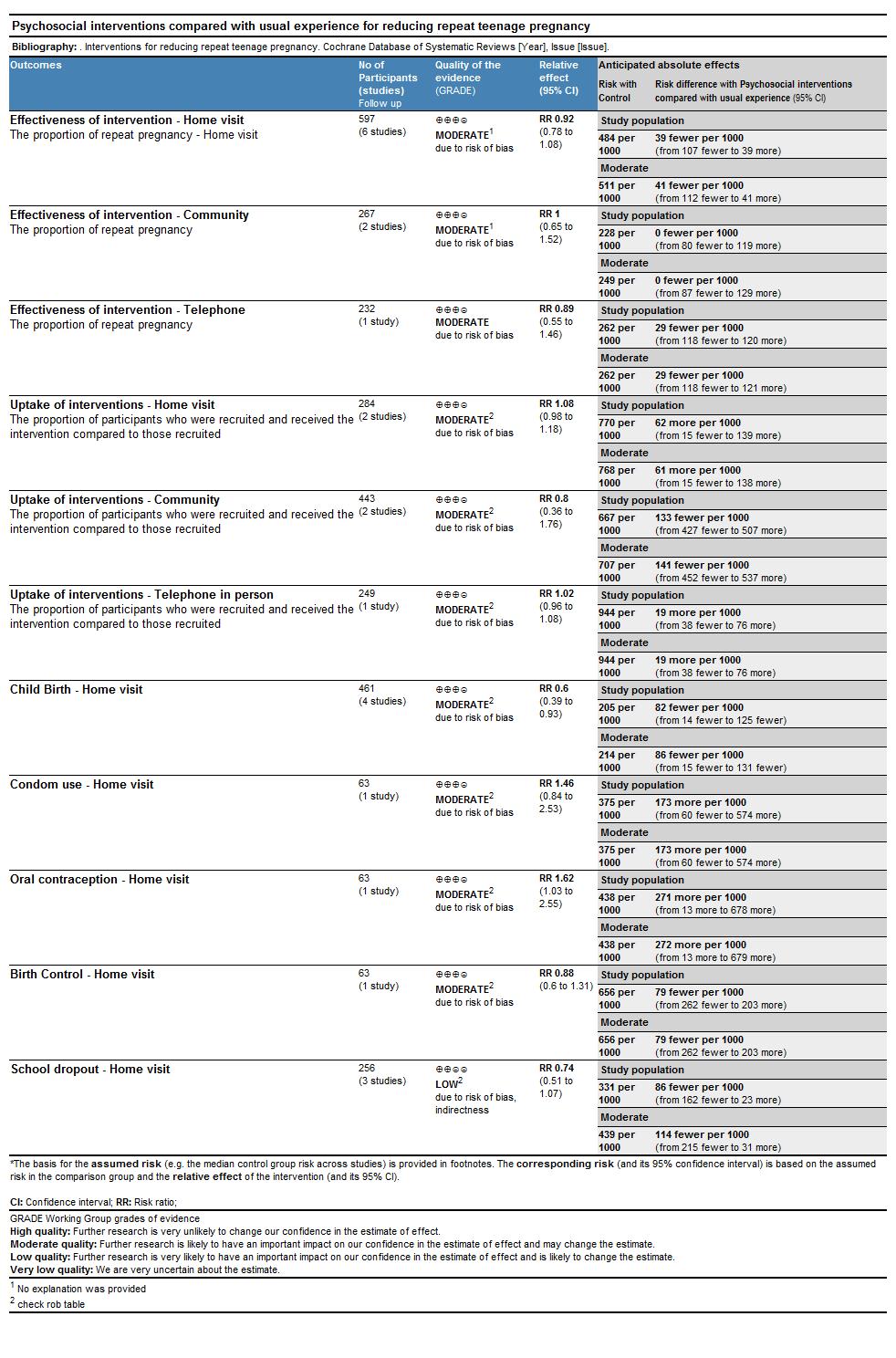


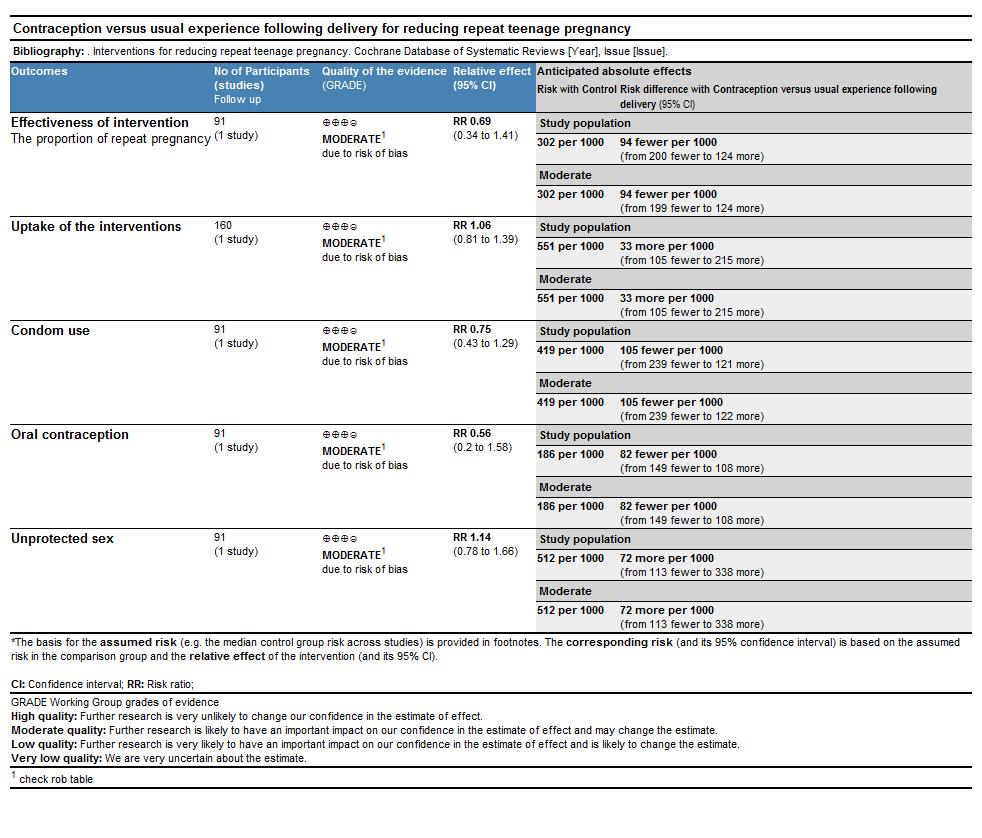


# Section 7, Summary of qualitative findings – CerQual profile

|  | **Summary statement** | **Certainty in the evidence** | **Explanation of the certainty in the evidence assessment** |
| --- | --- | --- | --- |
|  | In the context of unstable backgrounds, lack of family support, insecure housing, and chaotic lifestyles lacking in educational or vocational opportunities, there was no thought or planning; sexual activity was spontaneous and teenage pregnancy an accepted norm. | High certainty | The finding was from two moderate and high quality US studies, however the finding is plausible and likely to be transferrable to UK settings |
|  | Some repeat pregnancies did not result from chaotic lifestyles but were conceived to provide a sibling for the first-born or to complete childbearing before going back to education or training or employment. | High certainty | The finding was seen across two moderate and high quality US studies and one high quality UK study. |
|  | Some repeat pregnancies were conceived with the father in mind, either because he wanted a baby or because the mother felt it would help to consolidate the relationship. | High certainty | The finding was from three studies of moderate and high quality; two were from the US and one from the UK. |
|  | Some young mothers were determined not to get pregnant again and to manage their own sexual behaviour and contraception. | High certainty | The finding was from four studies of moderate to high quality. All four were conducted in the US but the finding is plausible and likely transferrable to UK settings. |
|  | Young mothers with goals and aspirations were less likely to have a repeat pregnancy; being a role model for their child was a motivating factor | High certainty | The finding was from three studies of moderate and high quality; two were from the US and one from the UK |
|  | Attempts to achieve educational or employment goals were frustrated by lack of childcare, inflexible school authorities and limited earning capacity in comparison with benefits. | High certainty | The finding was from one US and one UK study, both of high quality. |
|  | Lack of love, or the loss of a baby through miscarriage, abortion, still birth or being taken into care was a powerful motivator not to avoid a subsequent pregnancy | High certainty | The finding was from two high quality UK studies and one moderate quality US study |
|  | Being persuaded to have an abortion against a young woman’s wishes was traumatic and very likely to result in a rapid repeat pregnancy being kept secret to avoid repeating the experience. | High certainty | The finding was from two high quality UK studies. |
|  | Young women who made their own abortion decision and were supported were less likely to be traumatised or to regret their actions and more likely to take effective measures to avoid another pregnancy. | High certainty | The finding was only seen in one high quality UK study and one moderate quality Australian study but is plausible and likely to be transferrable to other settings. |
|  | Young women lacked basic understanding about fertility and knowledge about contraceptive methods. | High certainty | The finding was seen across six moderate to high quality US studies and one high quality study from the UK. |
|  | Young women enrolled in a school-based intervention had daily access to a public health nurse who provided information and with whom they felt comfortable in discussing contraception. | Low certainty | The finding was from one moderate quality US study and the supporting data for this finding were relatively thin. |
|  | A prevalent myth that deterred some young women from having an abortion, and led others to neglect to use contraception after having an abortion was that following an abortion a woman would never be able to conceive again. This misunderstanding largely derived from sex and relationships education in schools and from abortion clinic staff. | High certainty | The finding was from two high quality UK studies. |
|  | Timely contraceptive counselling and the provision to young mothers of the contraceptive method of their choice helped them to prevent a rapid repeat pregnancy. | High certainty | The finding was only seen in one moderate quality US study but nevertheless seems extremely plausible. |
|  | Inflexible appointment times and the inability of providers to counsel, prescribe and supply the method of choice in a single appointment were barriers to the timely uptake of effective contraception. | High certainty | The finding was from two high and moderate quality US studies and one high quality UK study. |
|  | Young women frequently changed contraceptive methods and this, along with the difficulty in accessing contraceptive services described above, led to gaps in protection | High certainty | The finding was from two US studies of moderate and high quality but seems plausible and likely to be transferrable to other settings |
|  | Some young women were deterred from using or discontinued using some types of contraception because of side effects, particularly weight gain or disruption of the menstrual cycle. | High certainty | The finding was seen in four US studies of moderate and high quality and seems likely to be transferrable to other settings. |
|  | Young women’s partners influenced their contraceptive choices and could support them (e.g. remind them to take the pill or persuade them to use LARC). However some wanted another baby and others objected to specific contraceptive methods (e.g. IUD). | High certainty | The finding was seen across five moderate and high quality studies. All were in the US but the finding seems likely to be transferrable to other settings. |
|  | Doctors or family members could also be supportive of contraceptive uptake and continued use. | Moderate certainty | The finding was from only one high quality US study but is likely to be transferrable to other settings. |
|  | Consistent use of oral contraceptives was a particular problem for young women with sometimes chaotic lifestyles and therefore many of them preferred LARC. | High certainty | The finding was seen in one high quality UK study, two moderate and high quality US studies, and one Australian study of moderate quality. |
|  | Some young women who had previously failed to use oral contraception consistently enough to prevent pregnancy still chose it after their baby was born. Some (but not all) found better ways of remembering to take it, e.g. a reminder alarm on their mobile phone. | Moderate certainty | The finding was from only one Australian study of moderate quality, but appears plausible. |
|  | For some young women even depo injections were unreliable because they entailed making and keeping regular doctor’s appointments; they preferred IUDs, which required minimum follow-up care. | Moderate certainty | The finding was from one high quality US study. |
|  | Young women enrolled in a school-based intervention had monthly pregnancy tests and a monthly questionnaire about their sexual activity and contraceptive use. They found this useful but complained of its intrusiveness. | Low certainty | This equivocal finding was from one moderate quality US study. |
|  | The idea of peer mentors had a mixed response. Some thought that, having had similar experiences, they might be more effective than older, more authoritative figures; others saw them as negative role models who had no right to preach. | Low certainty | This equivocal finding was from one high quality UK study.. |
|  | The provision of abortion services was patchy and access for vulnerable groups including teenagers was particularly difficult. | Moderate certainty | The study was from two high quality UK studies but both were set in London and might not be representative of services UK wide. |
|  | Among the young women interviewed in these studies there was a prevalence of negative, moralistic attitudes towards abortion that in some cases was related to religious or cultural beliefs but often appeared to have been influenced by the presentation of abortion in the context of sex and relationships education or religious education in schools. | High certainty | The study was from two high quality UK studies and one high quality US study. |

# Section 8, Sensitivity analysis forest plots

**Sensitivity analysis**

We conducted sensitivity analysis of the primary outcomes (unintended repeat Pregnancy & uptake of interventions) and secondary outcomes (Birth control/contraception & school out drop) including quasi-experimental and observational study.

We identified 3 quasi-experimental studies: Two reported the effectiveness and uptake of the interventions, and 1 reported on the acceptability of the intervention. We also identified 1 observational study which reported the effectiveness of the intervention, birth control/contraception and school out drop.

**Effectiveness of the interventions**

We added 3 studies in addition to 9 included in the primary analysis. Three of the four included studies, concerning 1484 teenagers, 709 receiving psychosocial interventions and 715 controls, reported the rate of unintended repeat pregnancy. All the reported participants were in the home visit subgroup.

By including additional studies, the results of the sensitivity analysis in Figure 1 show that in the home visit subgroup, the proportion of girls who experienced an unintended repeat teenage pregnancy was *further lower* compared to the primary analysis (home visit intervention (288/1077) compared with the control group (297/1004), with RR = 0.88 (95% CI 0.78 to 1;).

**
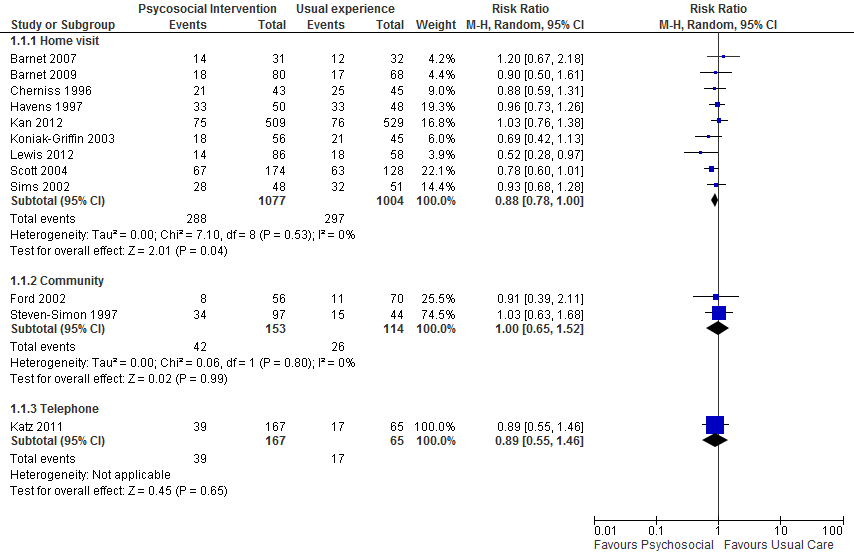
**

**Uptake of the interventions**

We added 2 studies in addition to 2 included in the primary analysis. Two of the four included studies, concerning 446 teenagers, 260 receiving psychosocial interventions and 186 controls, reported the rate of uptake of the intervention. All the reported participants were in the home visit subgroup.

The results of the sensitivity analysis in Figure 2 show that in the home visit subgroup, the proportion of girls who were recruited and received the intervention increased compared to the primary analysis (home visit intervention (280/405) compared with the control group (218/325), with RR = 1.07 (95% CI 0.99 to 1.16)


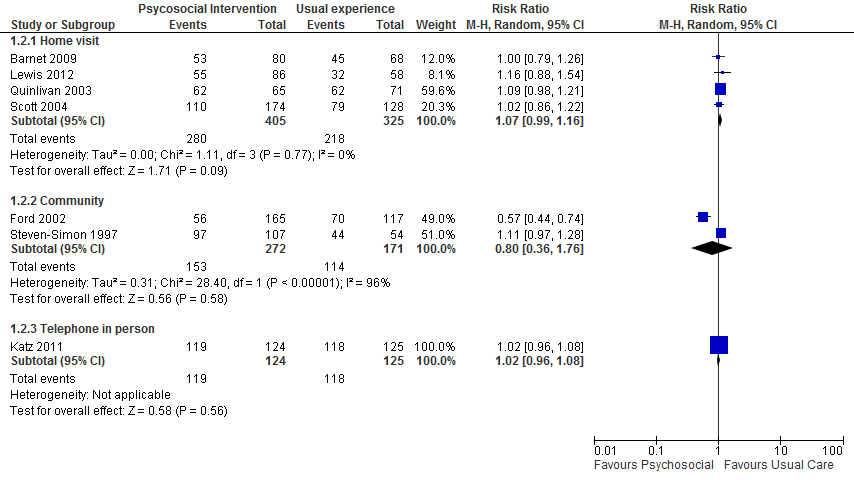


**Secondary outcomes**

**Birth control**


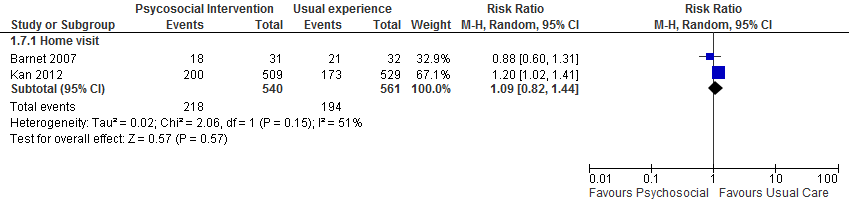


One study has been added in addition to 1 included in the primary analysis. The study, concerning 1038 teenagers, 509 receiving psychosocial interventions and 529 controls, reported the rate birth control. All the reported participants were in the home visit subgroup.

The results of the sensitivity analysis in Figure 3 show that in the home visit subgroup, the proportion of girls who were using birth control *shows a lower uptake rate in the control*  compared to the primary analysis (home visit intervention (218/540) compared with the control group (194/561), with RR = 1.09 (95% CI 0.82 to 1.44)

**School dropout**


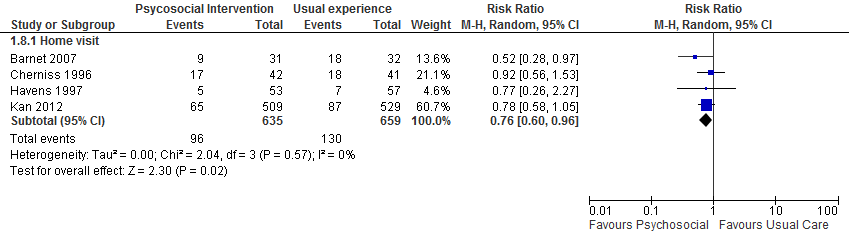


One study has been added in addition to 3 included in the primary analysis. The study, concerning 1038 teenagers, 509 receiving psychosocial interventions and 529 controls, reported the rate school dropout. All the reported participants were in the home visit subgroup.

The results of the sensitivity analysis in Figure 4 show that in the home visit subgroup, the proportion of girls who dropped school *is still in favour of psychosocial care* compared to the primary analysis (home visit intervention (96/635) compared with the control group (130/659), with RR = 0.76 (95% CI 0.60 to 0.96).

**Pregnancy**

Despite the negative findings, four studies Black Koniack barnet barnet reported childbirth (75 events from 461 participants). ‘Pregnancies’ covers all cases of known conception. These could have led to miscarriages, abortions, or still or live births. Three of the four studies reporting the childbirth gave RRs of < 1; however, none was significant. Appendix ? illustrates these data. When these results were combined in our meta-analysis, we found a RR of 0.60 (95% CI 0.39 to 0.93) indicating a significant reduction of the number of live births observed in the intervention arms of the studies.


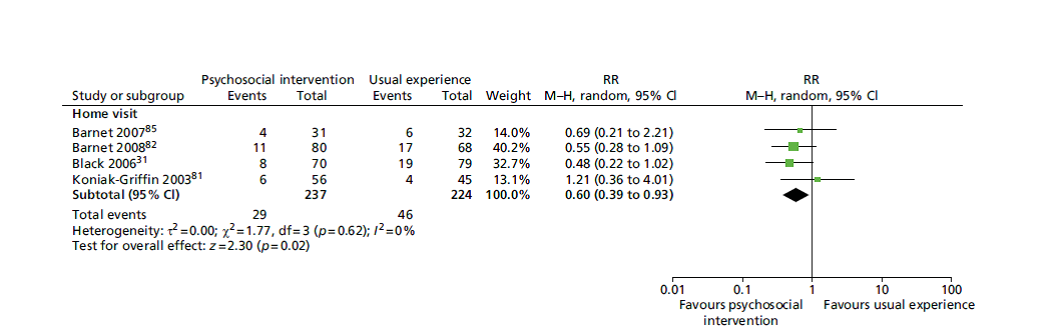


# Section 9, Realist Summary statements of emerging theory areas

**Motivation – Reasons for getting pregnant**

- Political View – the State’s view of teenage pregnancy
- Economic
- Family situation
- Question of unintended, unplanned or unwanted pregnancy or ambivalence

The likelihood of teenage pregnancy might be affected through paying attention to “Motivation” – in particular the reasons why a girl is pregnant, asking whether the pregnancy was unplanned, unintended, unwanted or whether the girl in question was ambivalent to motherhood and/or contraception use. Therefore interventions that pay attention to these factors may be more effective.

The likelihood of teenage pregnancy might be affected through paying attention to “State and Economic Views of Teenage Pregnancy.” The State (and media) tends to view teenage pregnancy as a negative phenomenon as teenage motherhood increases the likelihood of financial dependence on the state thus perpetuating the negative view; however, in certain cases teenage pregnancy can be a positive experience that does not result in financial dependence on the state.

**Connectedness**

- To peer group
- To family
- To school
- To the intervention itself
- To the person delivering the intervention
- Mentorship
- Personal care
- Collaborative decision making

The likelihood of teenage pregnancy might be affected through paying attention to ‘Connectedness' -positive/good relationships with family members, peers, their school and in certain cases the person delivering the intervention can provide a teenage girl with a sense of belonging and attachment to a group (which could in turn lead to feelings of empowerment). Therefore interventions that pay attention to these factors may be more effective.

**Targeting**

- Tailoring (including who delivers the intervention)
- Timing of the intervention
- Nature of the intervention e.g., single versus multi component
- Person focused rather than programme focused
- Collaborative decision making

The likelihood of teenage pregnancy might be affected through paying attention to ‘Tailoring’ - allowing for certain aspects of the intervention to be tailored e.g. timing, location and intervention focus can provide a personalised intervention that reduces barriers to attendance, increasing the likelihood of engagement and adherence to an intervention.

**Setting/Environment**

- Stigma
- “Norms” – society norm, family norm etc
- Partner influence
- Family setting
- Education setting
- Intervention setting

The likelihood of teenage pregnancy might be affected through paying attention to “Setting/Environment” – family and education setting may create a social norm in which teenage pregnancy is deemed an acceptable and common occurrence without stigma leading to a ‘normalisation’ of teenage pregnancy.

The likelihood of teenage pregnancy might be affected through paying attention in interventions to “Partner Influence” – a partner may have their own ideals on family size or in cases where the teenage girl in question has given birth to a previous partner’s child their current partner may their own desires to build a family with her and this may influence the teenage girl in both cases.

The likelihood of teenage pregnancy might be affected through paying attention to ”Intervention Setting” - providing the intervention in settings such as the home, community or school which minimise barriers to participation and attendance, increasing the likelihood of overall intervention engagement and continued attendance.

As part of the Cardiff Task and Finish Group meeting summary statements were prepared and presented for each theory area to explain the emerging themes of evidence to the attendees. The summary statement appears below

**Connectedness**

The likelihood of teenage pregnancy might be affected through paying attention to ‘Connectedness' - Adolescents need social support from parents, partners, friends and peers to develop their self-esteem, in order to increase their self-efficacy to reinforce their choices regarding the delay or avoidance of repeat pregnancy for example, accessing and consistent use of contraception. Adolescents without at least two people in their lives to verbalize their importance as a person or confirm their parenting skills were less likely to use birth control consistently. Interventions that provide social support through relatable sources to the mother such as peers, mentors or role models can assist to fill the gap in social support. Social support that builds trusting relationships can provide an adolescent mother with assistance, guidance, and confirmation of her self-worth. Interventions that use peers, mentors and role models may be more successful if they adopt a supportive “big sister” role, rather than an authoritarian “mother” “teacher” role. As many adolescents may have fractured relationships with their own mother, or conflicts with authority e.g., teachers, law enforcement.

**Intervention, Content**

The likelihood of teenage pregnancy might be affected through paying attention to ‘Intervention Content’ - Due to multiple levels of influence upon adolescents from families, peers and the community knowledge and information of contraception is not enough alone and often does not lead to implementation. Some have argued using social ecological theory to examine individual, dyad, family, peer/community and social system factors to develop intervention framework. The issue of adolescent pregnancy involves family, societal, medical, and educational components. Therefore, interventions should be multi-faceted providing a range of support and services that address the complex needs of young mums. Common components that have been consistently found in effective programs include: peer counselling, case management, school completion and career strategies and contraceptive availability.

**Intervention, Delivery**

The likelihood of teenage pregnancy might be affected through paying attention to ‘Intervention Delivery’ Interventions delivered in schools encourage a strong focus on remaining in education as the adolescent mother usually needs to be enrolled in school to access these services and interventions. Engagement in school based activities has been found to be a protective factor against pregnancy in adolescence. This could see a shift in resources in schools from downward stream, reactive interventions to more upstream, proactive interventions that aim to prevent an initial pregnancy in adolescence rather than repeat pregnancies in adolescence. The dichotomy between the access and availability of services for urban versus rural adolescents was also evident; stating a need for better access in rural areas.

**Perspective/Context**

The likelihood of teenage pregnancy might be affected through paying attention to ‘Perspective/Context’ Family dynamics, past experiences and expectations of gender roles can influence an adolescent girl’s perceptions of motherhood. Interventions should be sensitive to these personal experiences when developing and delivering interventions for example, an adolescent who has experienced multiple miscarriages may experience feelings of guilt, loss and depression leading to thoughts of repeat pregnancy to replace the baby or babies they have lost. If an intervention pays attention to this history and its potential effect on decision making, the appropriate counselling may be delivered as a part of the intervention allowing the adolescent to deal with these feelings of loss without resulting in a repeat pregnancy.

**Other Goals and Aspirations**

The likelihood of teenage pregnancy might be affected through paying attention to ‘Other goals and Aspirations’ Other goals and aspirations outside of motherhood may help teenagers equate consistent contraception use with obtaining the lifestyle they want. Without other alternatives motherhood may become the only option to provide girls with an opportunity for success and autonomy. However, if the adolescent is already a mother there is a need for flexibility and additional support to help these girls achieve their goals through intervention tailoring and structure for example, extended deadlines for school or college course work or graduation requirements. Interventions that provide a range of services such as birth control, social support, service co-ordination, health education, life skills and employment training may equip adolescents with the skills they need to seek out alternative life choices.

**Perceptions of Parental Responsibility**

The likelihood of teenage pregnancy might be affected through paying attention to ‘Perceptions of Parental Responsibility’ Adolescent mothers report the desire to provide a better upbringing for their own child than they experienced, they see the opportunity to rectify the mistakes made in their own childhood. There is also a difficulty in adolescent mothers developing autonomy and self-sufficiency in parenting, as maternal grandmothers often under-mine the adolescent’s self-sufficiency by taking over the parenting role. This clash for parental responsibility can lead the adolescent to a repeat pregnancy in order to gain autonomy and the sole parenting role of a child, as the grandparent is now needed to parent the older sibling/s. Interventions should aim to support self-sufficiency equipping parents with the skills they need to be the capable, competent parents they wish to be.

**Setting/Environment Individual**

The likelihood of teenage pregnancy might be affected through paying attention to ‘Setting/Environment Individual’ Personal characteristics and circumstances influence an adolescent girl’s ideas about the likelihood, implications and potential benefits to becoming a mother during adolescence. The personality of an adolescent is important. There are sexually active adolescents who do not become pregnant. It has been suggested that exploring the characteristics and personalities of never-pregnant, sexually active adolescents to uncover exactly why they do not get pregnant, despite sexual activity could provide insight into effective interventions. For example, do these never-pregnant sexually active adolescents feel they can speak openly about sex with mothers, friends, and partners? Do they feel the personal cost of stigma when accessing emergency contraception is less than the personal cost of a pregnancy during adolescence?

**Tailoring**

The likelihood of teenage pregnancy might be affected through paying attention to ‘Tailoring’ There are multiple levels of influence upon an adolescent and thus interventions should be geared towards an adolescent’s readiness to change rather than standardised messages and advice. Interventions that pay attention to adolescent’s life experiences as well as their developmental stage, cultural context, age-appropriate impulsive and rational decision-making styles and responses to stress may be more successful than interventions based upon a medical model. Medical models of interventions are defined as interventions that focus mainly on information and access of contraception only, an example of this type of model is the suggestion that the most effective way to help adolescent mothers might be to encourage them to use long acting injectable or implant contraception methods. Interventions should also be sensitive to barriers including practical, emotional and psychological barriers and develop an intervention that can overcome these barriers for example, practical issues of access by providing interventions at home, or transport if the intervention is to be delivered in another setting such as a community centre. Interventions also need to understand adolescent sexual behaviour from the point of view of the adolescents themselves. Many interventions are designed based upon adult beliefs of adolescent sexual activity and the consequences of adolescent pregnancy, which may lack relevance to the adolescent population the intervention aims to serve. Interventions should be modelled using adolescent principles and perceptions of sex and relationships and the potential consequences of pregnancy during adolescence.

These findings resonated with attendees at the stakeholder group, the stakeholders only wished to add with regards to tailoring they felt interventions needed to be tailored to the individual according to their circumstances at the time. They said “girls complain that they are not listened to by professionals”. They also stated “we really need to find out what they really need and want, and understand what they are asking for – there is too much generalisation, and perceptions of what girls want are not accurate”.

# Section 10, Overarching synthesis table

| Intervention component | Programme theory/ logic | Risk factors | Stakeholder views and implementation issues | Realist Theory Area | Stakeholder feedback | Service Users’ feedback |
| --- | --- | --- | --- | --- | --- | --- |
| *Randomised Controlled Trials* | | *Meta-regression and qualitative evidence* | *Qualitative evidence* | *Interpretation of the evidence base* | *Indirect evidence* | *Indirect evidence* |
| Computer Assisted Motivational Interviewing (CAMI) ^58,59^ | MI emphasizes personal goals and self-efficacy and addresses any discrepancies between them thereby refocusing on goals that are incompatible with repeat pregnancy | Lack of goals/self-efficacy was not an identified risk factor | Young mothers with goals and aspirations were less likely to have a repeat pregnancy. | Tailoring (1)  Connectedness (1)  Perspective/ Context | Utilizing MI techniques to make every contact matter by GP, midwife, health visitors count | Preference for home-visits since it felt personal with more space to discuss things that could not be discussed in-group. Practical preparation time for child (dressed, fed, nappy changed) and self was short. |
| CAMI + monthly home visiting by para-professional^58,59^ | Home visits by professionals lead to feelings of support and encouragement of life choices support and the perception that others are encouraging life choices. | Lack of life choices was not an identified risk factor | There was no evidence relating to home visits in any of the qualitative studies | Tailoring (1)  Connectedness (1)  Perspective/ Context | Home visits are more likely to be useful than interventions that rely on young women travelling to a clinic. |  |
| Weekly home visiting by para-professional^24^ | Repeated home visits remove barriers to accessing services and are designed to address gaps in social support networks and sustain behaviour change by repeated contact to direct young women away from repeat pregnancy. | Lack of access to services or social support were not identified as risk factors | There was no evidence relating to home visits by professional, para-professional or lay persons | Tailoring (2) Connectedness (1)  Perspective/ Context |  |  |
| Trained volunteer mentors to give telephone support and social outings  12 hours per month^60^ | Using the mentor as a role model, information/ education resource and a source of social support for young mother and her family could reduce repeat pregnancy | Lack of social support was not an identified risk factor, but living with a partner was weakly associated with an increase in repeat pregnancy | There was no evidence relating to either trained mentors or role models, however there was evidence that some young women perceived a negative stereotype of young motherhood, which they were determined not to conform to | Tailoring (1)  Connectedness (1)  Perspective/ Context |  |  |
| Small group educational intervention to promote antenatal care and well-baby care([94](#_ENREF_94))^23^ | A peer support group prevents unplanned repeat pregnancies by addressing self-efficacy and improving self-concept amongst young mothers. | Lack of peer support and self-efficacy were not identified as risk factors, but continued education was weakly associated with a reduction in repeat pregnancies | In some contexts pregnancy in young women was perceived to be the norm. | Connectedness  (1, 3)  Perspective/ Context | A “buddy” system or peer support group could offer choices, empower young women, give them confidence as well as create space for them to state what they want and need. | The young mothers enjoyed taking part in a group as they can see everyone, hear everyone’s opinion, and know that they are not alone. |
| Weekly supportive group meetings and monthly pregnancy test^61^ | A peer support group helps build trusting relationships and prevents unplanned repeat pregnancies by addressing self-efficacy and improving self-concept amongst young mothers. | Lack of peer support was not identified as a risk factor | There was no evidence relating to organised group support. Informal peer group influence tended to reinforce a view of parenthood in young women as the social norm | Connectedness (3)  Perspective/ Context |  |  |
| Monetary incentive + weekly supportive group meetings and monthly pregnancy test^61^ | Monetary incentives seek to improve attendance for a peer support group that helps build trusting relationships and prevents unplanned repeat pregnancies by addressing self-efficacy and improving self-concept amongst young mothers. | Finance was not an identified risk factor | There was no evidence regarding monetary incentives in this context | Tailoring (3)  Connectedness  (1, 3)  Perspective/ Context |  |  |
|  |  |  |  |  |  | Transport to and from the location and the availability of food, refreshments and crèche facilities can all increase engagement and improve attendance rates |
| Monetary  incentive and  monthly pregnancy test^61^ | The monetary enticement designed to improve group attendance so in the absence of the group component the monetary incentive should have little effect on pregnancy avoidance | Finance was not an identified risk factor | Some young mothers in the UK complained of inadequate provision of child care or financial support to allow them to continue their education or take up a job. Time spent with their children was of higher value to them than the marginal difference in income between benefits and what they could earn from low-paid work | Tailoring (3)  Perspective/ Context |  |  |
| 42 telephone counselling sessions up to 18 months postpartum by Masters level young women following a manual based written curriculum^29^ | Healthy communication skills and connectedness with community can influence repeat pregnancy by reducing negative behaviours through knowledge expansion and behaviour change. | Lack of support and knowledge were not identified risk factors | There was no evidence relating to telephone counselling in any of the included qualitative studies. However some young women lacked basic knowledge about fertility and contraception | Tailoring (1)   Connectedness (3)  Perspective/ Context |  |  |
| Trained professional home visitors visited bi-weekly for one year and monthly for second year and delivered parenting skills, safe sex, pregnancy prevention and goal setting^62,63^ | Home visiting aims to reduce rates of repeat pregnancy through education and support and may be suitable for adolescents who are hard to engage, at high risk and living under adverse conditions. | Lack of pregnancy prevention skills was not an identified risk factor. However, evidence that LARC, oral, or other types of contraceptive reduced repeat pregnancy was weak; education and support in the appropriate and consistent use of contraceptives could have been lacking | There was no evidence relating to professional home visiting in any of the included qualitative studies. However some young women lacked basic knowledge about fertility and contraception | Connectedness (3)  Perspective/ Context | Home visits are more likely to be useful than interventions that rely on young women travelling to a clinic. | Preference for home-visits since it felt personal with more space to discuss things that could not be discussed in group, and also helps to adjust to demands of motherhood. Practical preparation time for child (dressed, fed, nappy changed) and self was short. |
| Peer mentors (“big sisters”) delivered 19 lesson home based intervention with parenting skills, pregnancy prevention and condom provision^43^ | The use of support and self-efficacy through peer mentors can influence repeat pregnancies by emphasizing goal setting and involving family members at home. | Lack of self-efficacy and support were not identified risk factors | In the context of unstable backgrounds, chaotic lifestyles and lack of family support, sexual activity was spontaneous and without thought. There was also a lack of basic knowledge about fertility and contraception | Tailoring (1)   Connectedness  (1, 3)  Perspective/ Context |  |  |
| Nurse midwives delivered 6 home visits for first 6 months postnatal; childcare, parenting skills, contraceptive advice^48^ | Repeated home visits remove barriers to accessing services for improving care for young mothers and infants sustain behaviour change by repeated contact to direct young mothers away from repeat pregnancy. | Evidence that contraceptive use reduced repeat pregnancies was weak; appropriate access, skills and knowledge to support consistent use of contraception could have been lacking | Inflexible appointment times and the inability of providers to promptly supply the contraceptive of choice led to gaps in contraceptive protection postpartum and when young women decided to change methods. | Connectedness  (1, 3) Tailoring (2)  Perspective/ Context |  |  |
| Advance supply of emergency contraception^48^ | Supplying emergency contraception is aimed at reducing repeat pregnancies by addressing frequent discontinuation or switching of contraceptive methods. | Evidence that contraceptive use reduced repeat pregnancies was weak, but inconsistency of use was not investigated as a risk factor | Many young women experienced unwanted side-effects of contraception, unfortunately often associated with more reliable methods. Stopping one method before obtaining another was not uncommon and left them vulnerable to unwanted pregnancy in the period between. | Tailoring (2)  Perspective/ Context | Emergency contraception is not the answer for girls with chaotic lifestyles as there is a danger that they would not use it as intended but would rely on it as their sole method of contraception. Since LARCs are not easily accessible through GPs, or repeated appointments have to be made, it increases susceptibility to repeat pregnancy. | The overlap of the 72 hour requirement of the emergency contraception and Bank Holiday or Christmas is a cause of concern. There was also hesitancy in asking or acquiring for the emergency contraception for fear of being judged. Preference for LARC as well, since it takes care of things for a long time. |
| Intensive family based therapy home visits, developmental guidance, supportive counselling^22^ | Repeated home visits by a profession involving other family members can improve engagement with dysfunctional families direct young mothers away from repeat pregnancy | Poor family relationships were not identified as a risk factor; history of abuse was not significantly associated with repeat pregnancy | In the context of unstable backgrounds, chaotic lifestyles and lack of family support, sexual activity was spontaneous and without thought. | Tailoring (1, 2)  Connectedness  (1, 2, 3)  Perspective/ Context | Home visits are more likely to be useful than interventions that rely on young women travelling to a clinic. | The decision to complete a family in the present than later could influence decision to forgo birth spacing.  Partner abuse (some like to keep them pregnant) and sexual exploitation by older men targeting young looked-after girls in the name of having a ‘family’ could form the basis for repeat pregnancies.  Absence of Life skills training makes young mothers more susceptible to repeat pregnancies  Preference for home-visits etc. |
| The individual treatment condition received home visits, developmental guidance, supportive counselling, and help in acquiring other needed services^22^ | Repeated home visits remove barriers to accessing services for improving care for young mothers and infants sustain behaviour change by repeated contact to direct young mothers away from repeat pregnancy. | Lack of support and access to services were not identified risk factors | There was no evidence relating to home visits in any of the included qualitative studies. But some young women complained of poor access and a lack of continuity in contraceptive care. Others were deficient in basic knowledge about fertility and contraception | Connectedness (3)  Perspective/ Context | Home visits are more likely to be useful than interventions that rely on young women travelling to a clinic. | Preference for home-visits since it felt personal with more space to discuss things discussed in group, and also helps to adjust to demands of motherhood. Practical preparation time for child (dressed, fed, nappy changed) and self was short. |
| Cluster randomised controlled trial |  |  |  | Interpretation of the evidence base | Indirect evidence | Indirect evidence |
| Monthly individual contacts on campus and five yearly home visits + case management with state wide teaching component with additional curriculum details^64^ | Repeated home and school visits remove barriers to accessing services and are designed to address gaps in social support networks and sustain behaviour change by repeated contact to direct young women away from repeat pregnancy. | The evidence that remaining in education reduced repeat pregnancies was inconclusive | There was no evidence relating to home or school visits in any of the included qualitative studies. However, some young women lacked basic knowledge about fertility and contraception and had poor access to contraceptive care. | Connectedness  (1, 3)  Tailoring (1, 2)  Perspective/ Context | Home visits are more likely to be useful than interventions that rely on young women travelling to a clinic. | Preference for home-visits since it felt personal with more space to discuss things that could not be discussed in group. Practical preparation time for child (dressed, fed, nappy changed) and self was short. |
| Non randomised trials |  |  |  | Interpretation of the evidence base | Indirect evidence | Indirect evidence |
| Relationship-focused, intensive case management program with bimonthly contacts for up to 3 years^46,65^ | Repeated home visits by a mentor aimed at developing resilience could reduce repeat pregnancy by a) developing personal goals and self-efficacy, and b) using the mentor as a role model, information/ education resource and a source of social support for the young mother. | Lack of support was not an identified risk factor | There was no evidence relating to intensive case management in any of the included qualitative studies. There was a lack of continuity of care including contraceptive care and post-abortion care. | Connectedness  (1, 3)  Tailoring (1)  Perspective/ Context |  |  |
| Simple case management services^46,65^ | Using the mentor as a role model, information/ education resource and a source of social support for young mother could reduce repeat pregnancy. | Lack of support was not an identified risk factor | There was no evidence relating to simple case management in any of the included qualitative studies | Connectedness  (1, 3)  Tailoring (1) |  |  |
| Family planning methods by approaching beliefs about health *(Health Belief Model)*^66^ | Adherence to family planning methods may be influenced by addressing the personal beliefs or perceptions about health behaviours of young mothers. | Health beliefs were not an identified risk factor | There was no evidence relating to family planning from a health belief perspective; failures in effective contraceptive use was attributed to lack of knowledge, understanding or access | Tailoring (1)  Perspective/ Context | The need to give each partner a child as a token of commitment. |  |
| Family planning methods by approaching social contexts in which health/illness exists *(PRECEDE*)^66^ | Adherence to family planning methods may be influenced by understanding the social context in which repeat pregnancy exists. | Social contexts were not considered in terms of their relationship to contraceptive use | Repeat pregnancies were more likely to occur in the context of unstable backgrounds, lack of family support, insecure housing, and chaotic lifestyles lacking in educational or vocational opportunities, when there was no thought or planning; sexual activity was spontaneous and pregnancy in young women an accepted norm. | Tailoring (1) | Contraception not portrayed in sex scenes in films.  Social media encourages casual relationships. Partner abuse (some like to keep them pregnant) and sexual exploitation by older men targeting young looked-after girls in the name of having a ‘family’ could form the basis for repeat pregnancies. Absence of Life skills training makes young mothers more susceptible to repeat pregnancies. |  |

In that matrix, we have adopted the following colour coding to illustrate theories addressed by the studies,

Social cognitive ecological theory

Developmental theories

Resilience theory

Resilience-recoil-rebound theory

Recoil-rebound theory

In addition, we have summarised the discussion of the Realist Theory areas in the following way:

**Tailoring (1)** - Paying attention to adolescent’s life experiences as well as their developmental stage, cultural context, age-appropriate impulsive and rational decision-making styles and responses to stress.

**Tailoring (2)** - By reducing practical barriers to the intervention such as home visits or accessing emergency contraception at clinics or pharmacies may increase engagement

**Tailoring (3)** - Offering incentives to increase engagement and attendance

**Connectedness (1)** - Feelings of support and being connected to the professional may trigger feelings of self-determination and active control. Self-efficacy, self-esteem and empowerment.

**Connectedness (2)** - Feeling connected and supported helps adolescents feel their life choices are being encouraged

**Connectedness (3)** - Feelings of support and being connected to the group may trigger feelings of ‘being heard'

**Perspective/ Context** - Exploring motivations (e.g., norms, peers and past experiences) to lead to better engage with the issue of sex and pregnancy.

1. This adds up to 27 (not 26) but is as reported in both paper and thesis [↑](#footnote-ref-1)
